# Electronic and Optical Properties of Eu^2+^-Activated Narrow-Band Phosphors for Phosphor-Converted Light-Emitting Diode Applications: Insights from a Theoretical Spectroscopy Perspective

**DOI:** 10.1021/jacs.2c00218

**Published:** 2022-04-26

**Authors:** Rami Shafei, Dimitrios Maganas, Philipp Jean Strobel, Peter J. Schmidt, Wolfgang Schnick, Frank Neese

**Affiliations:** † Max-Planck-Institut für Kohlenforschung, Kaiser-Wilhelm-Platz 1, Mülheim an der Ruhr 45470, Germany; ‡ Department of Chemistry, University of Munich (LMU), Butenandtstraße 5-13, München 81377, Germany; § Lumileds Phosphor Center Aachen, Lumileds (Germany) GmbH, Philipsstraße 8, Aachen 52068 , Germany; ∥ Department of Chemistry, Faculty of Science, Beni-Suef University, Salah Salem Str, Beni-Suef 62511, Egypt

## Abstract

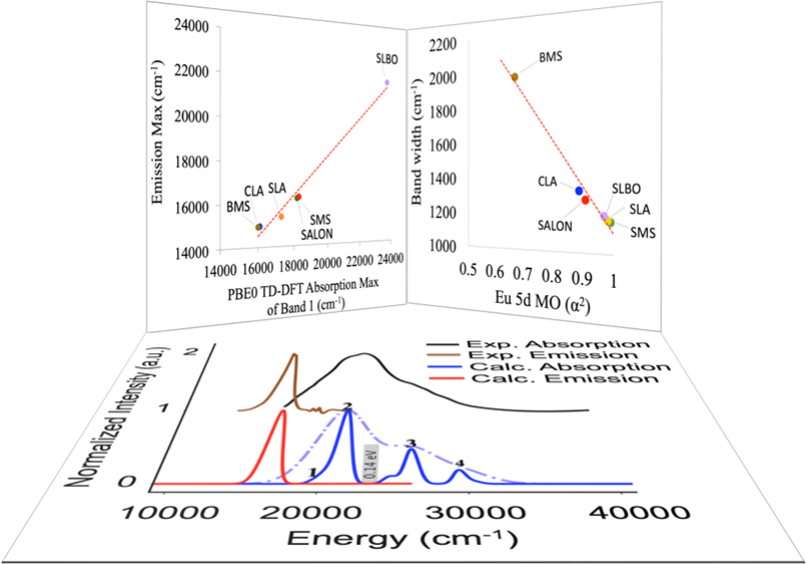

In this work, we present a computational protocol that is able to predict the experimental absorption and emission spectral shapes of Eu^2+^-doped phosphors. The protocol is based on time-dependent density functional theory and operates in conjunction with an excited-state dynamics approach. It is demonstrated that across the study set consisting of representative examples of nitride, oxo-nitride, and oxide Eu^2+^-doped phosphors, the energy distribution and the band shape of the emission spectrum are related to the nature of the 4f–5d transitions that are probed in the absorption process. Since the 4f orbitals are very nearly nonbonding, the decisive quantity is the covalency of the 5d acceptor orbitals that become populated in the electronically excited state that leads to emission. The stronger the (anti) bonding interaction between the lanthanide and the ligands is in the excited state, the larger will be the excited state distortion. Consequently, the corresponding emission will get broader due to the vibronic progression that is induced by the structural distortion. In addition, the energy separation of the absorption bands that are dominated by states with valence 4f–5d and a metal to ligand charge transfer character defines a measure for the thermal quenching of the studied Eu^2+^-doped phosphors. Based on this analysis, simple descriptors are identified that show a strong correlation with the energy position and bandwidth of the experimental emission bands without the need for elaborate calculations. Overall, we believe that this study serves as an important reference for designing new Eu^2+^-doped phosphors with desired photoluminescence properties.

## Introduction

Over the last few decades, light-emitting diode (LED) phosphors have been widely used in applications in the field of modern lighting and display technologies. In particular, phosphor-converted LED (pc-LED) materials provide lighting sources that (1) show high luminous efficacy and (2) operate under low power consumption and (3) they are environmentally friendly.^1−5^ Due to these reasons, pc-LEDs are considered as the next-generation solid-state lighting technology with the potential to substitute traditional lighting sources ranging between incandescent, fluorescent, and halogen lamps to backlights of liquid crystal displays (LCDs).^6,7^

Successful design of innovative materials featuring pc-LED phosphors requires (1) a high degree of tunability of the emission band position in the range between red and blue light, (2) a narrow band width of the emission band, and (3) thermal stability.^1,2,6,8−21^ In fact, a high degree of tunability implies that the targeted pc-LED phosphors show systematic geometric and electronic structure properties.^2,9,14,15,22−24^ Hence, identification of such systematic descriptors is of paramount importance for the design of pc-LED phosphors with desired luminescence properties.

This has led to the development of combined experimental and theoretical protocols that are aiming to establish at least semiquantitative rules that relate the geometric and electronic structure of the system under investigation to the desired spectroscopic properties of such materials. A number of correlations have been attempted on the basis of density functional theory (DFT) calculations that relate properties such as the crystal field strength or the band gap to the Stokes shift of classes of pc-LED phosphors.^9,14,15,23−28^ Machine learning techniques^8,29^ have also been employed in an effort to provide predictions of novel materials with the desired properties summarized above. While the usefulness of DFT^8^ or machine learning-based approaches alongside empirical correlations^29^ is undeniable, they also suffer from some significant shortcomings that will be elaborated on below. In fact, to date, most promising phosphors are still discovered by an elaborate trial-and-error process. Thus, there is a continued desire to improve the predictive power of the theoretical protocols to better aid the design of new phosphors with tailored properties. This paper represents an attempt in this direction.

Combining a range of spectroscopic methods with the results of carefully calibrated theoretical spectroscopy has been widely used on the fields of (bio)inorganic chemistry and catalysis.^30−37^ It has been indeed shown that this approach has been proven instrumental to characterize exotic reactive species in model systems (e.g., the genuine Fe(V)^35^ and the first Fe(VI)^36^ centers in coordinate complexes), clarifying the structure and oxidation states in enzymes, for example, the identification of a unique carbide center in the active site of the dinitrogen-activating enzyme nitrogenase^34^ or the characterization of the structure and oxidation states of the oxygen-evolving complex in photosynthesis.^30,32^ Moreover, this approach has shown great potential in bridging the worlds of homogeneous and heterogeneous catalysis by interpreting simultaneously the spectroscopic response of vanadium-based oxidation catalysts.^38^ It should be emphasized that in order to successfully apply the theoretical methodologies to interpret the spectroscopic response of real-life chemical problems, it is of paramount importance that the theoretical methods are properly calibrated, implying that the error bars of the theoretical predictions must be known from studying a series of known and understood systems. Hence, in all the above-described cases, all predictions of the theoretical methods are only valid within the confidence intervals defined by the calibration procedure.^39^

In this work, we will use high-level wavefunction-based *ab initio* quantum chemistry in conjunction with time-dependent density functional theory (TD-DFT) and an excited-state dynamics (ESD) approach to show that a carefully calibrated multimethod protocol can be developed that is able to quantitatively predict the spectroscopic properties of pc-LED phosphors in terms of energy position and the bandwidth of the targeted emission spectrum. However, in addition to producing prediction of near quantitative accuracy, it is possible to extract more qualitative data from the calculations that can serve to guide chemical intuition in the design of new and improved systems based on geometric and electronic structure properties such as the crystal field strength, the band gap, and the Stokes shift of the investigated phosphor. For this purpose, a selection of Eu^2+^-doped phosphors in nitride/oxo-nitride/oxide inorganic hosts is chosen. These phosphors adopt a UCr_4_C_4_-type or related crystal structure^40^ and exhibit promising luminescence spectral features, such as narrow-band emission, with a high degree of tunability that is offered by the host structure. In a first step, a multimethod theoretical protocol will be developed that is able to predict the bandwidth and the energy position of the absorption and fluorescence spectra of four nitride phosphors Sr[Mg_3_SiN_4_]:Eu^2+^ (SMS),^16^ Ba[Mg_3_SiN_4_]:Eu^2+^ (BMS),^11^ Ca[LiAl_3_N_4_]:Eu^2+^ (CLA),^12^ and Sr[LiAl_3_N_4_]:Eu^2+^ (SLA).^13^ In a second step the above protocol will be employed to study the electronic structure and the spectroscopic response of narrow-band oxo-nitride red phosphor Sr[Al_2_Li_2_O_2_N_2_]:Eu^2+^ (SALON)^41^ and the ultranarrow-band blue oxide phosphor SrLi_2_[Be_4_O_6_]:Eu^2+^ (SLBO).^1^ In a final step, a homogeneous set of descriptors will be extracted from the developed computational protocol that is able to predict the emission energy position and the bandwidth of Eu^2+^ phosphors in an attempt to aid the synthetic efforts toward new phosphors with desired photoluminescence properties.

## Experimental/Methods

### Study Set: Geometric Structure

**Figure 1:**
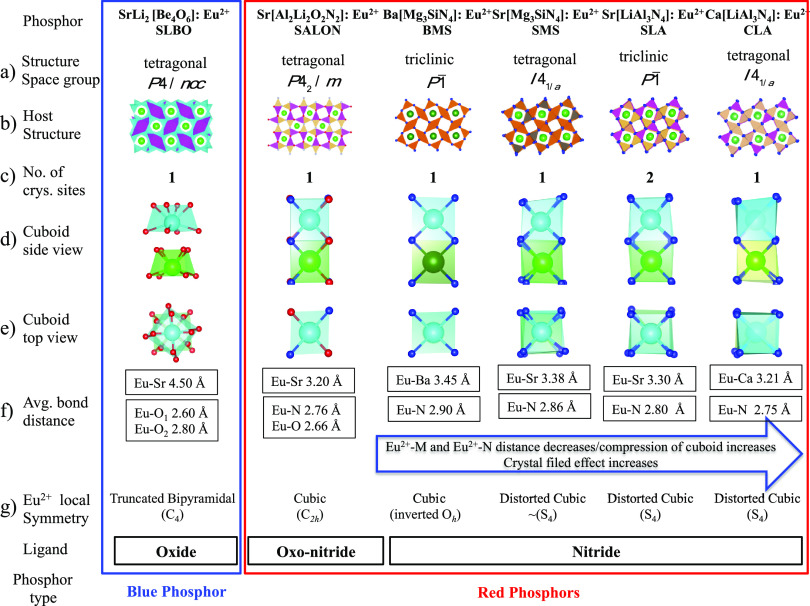
Atomic structures of red SMS, SLA, CLA, BMS, SALON, and blue SLBO phosphors. (a, b) Graphical representation of phosphor host crystal structures together with their crystallographic space groups. (c) Number of crystallographic sites for Eu^2+^. (d, e) Side and top view of cuboids containing the two adjacent EuL_8_ and ML_8_ cuboids (M = Ca, Sr, Ba, L = N, O). (f) Most important bond lengths Eu–M and Eu–L. (g) Local symmetry around the Eu/ML_8_ centers. Atom colors: Ca (light green), Sr (green), Ba (deep green), Eu (cyan), Si (gray), Mg (orange), Al (yellowish pink), Li (pink), N (blue), O (red).

The chosen study set of phosphors is presented in Figure 1 together with the major structural characteristics of the different coordination environments around the doped Eu^2+^ centers.

As seen in Figure 1, the selected nitride phosphors contain an ordered distribution of edge- and corner-sharing (Si,Mg)N_4_ or (Al,Li)N_4_ tetrahedral building units, which form vierer ring channels. The host M^2+^ ions (M = Ba, Sr, Ca) as well as the doped Eu^2+^ ions are placed at the center of those vierer ring channels, and they are 8-fold coordinated by nitride N^3–^ ions. In all the cases, the symmetry is lower than cubic; as a result, cuboid-like polyhedra Eu/ML_8_ are formed (for simplicity, these polyhedra will be referred to as cuboids). In particular, the nitridomagnesosilicate phosphors M[Mg_3_SiN_4_]:Eu^2+^ (M = Sr (SMS), Ba (BMS)) crystallize in an ordered variant of the UCr_4_C_4_ structure type. SMS crystallizes in a tetragonal crystal structure with space group *I*4_1_/*a*, isotypic to Na[Li_3_SiO_4_] with the local symmetry of the Eu/SrL_8_ coordination polyhedra being distorted cubic. On the contrary, BMS is a distorted variant of the UCr_4_C_4_ structure type as it crystallizes in triclinic space group *P*1̅. The tetragonal distortion reduces the local symmetry of the Eu/SrL_8_ to approximately *S*_4_. Similarly, the nitridolithoaluminate phosphors M[LiAl_3_N_4_]:Eu^2+^ (M = Sr (SLA), Ca (CLA)) are also tetragonally distorted variants of the UCr_4_C_4_ structure type crystallizing in triclinic *P*1̅ and tetragonal *I*4_1_/*a* space groups, respectively. As in the case of SMS, in SLA and CLA, the Eu/SrL_8_ and Eu/CaL_8_ centers are tetragonally distorted (e.g., they can be approximated by *S*_4_ symmetry). Along the BMS, SMS, SLA, and CLA sequence, the crystal field strength, as well as the Eu/ML_8_ cuboid compression, is increasing.^42^ This is reflected by the decrease of the Eu-M (M = Ba, Sr, Ca) (from 3.45 to 3.21 Å) and Eu-N (from 2.90 to 2.75 Å) bond lengths, respectively, across the sequence. The oxonitridolithoaluminate phosphor Sr[Li_2_Al_2_O_2_N_2_]:Eu^2+^ (SALON) is also an ordered variant of the UCr_4_C_4_ structure type that crystallizes in the tetragonal space group *P*4_2_/*m*. Two kinds of tetrahedra ([AlON_3_]^8–^ and [LiO_3_N]^8–^) are now forming a condensed network of tetrahedra leading to three different types of channels along the [001] direction. The channel hosting the Sr^2+^ or the doped Eu^2+^ cations forms Eu/SrN_4_O_4_*C*_2*h*_ symmetric cuboids with Sr–N of 2.76 Å, Sr–O of 2.66 Å, and Eu–Sr of 3.21 Å bond lengths. These structural characteristics are very similar to the CLA nitride phosphor as will be discussed below. Finally, the oxoberyllate phosphor SrLi_2_[Be_4_O_6_]:Eu^2+^ (SLBO) crystallizes in the space group *P*4/*ncc* and contains edge- and corner-sharing BeO_4_ tetrahedra, which are forming two kinds of vierer ring channels along the [001] direction. The channel hosting the Sr^2+^ or the doped Eu^2+^ ions forms Eu/SrO_8_*C*_4_ sequences of truncated bipyramidal cuboids in which the individual Eu/SrO_8_ cuboids are rotated by 45° with respect to each other. The Eu–O bond lengths range between 2.6 and 2.8 Å while the Eu–Sr bond lengths are quite elongated (4.5 Å). This is due to the fact that the rotated pairs of Eu/SrO_8_ cuboids do not share common faces, as in all the other selected phosphors. To show that our new method can be applied on phosphors comprising the entire visible spectrum, and having a structure that is not related to the UCr_4_C_4_ type, a blue phosphor was additionally selected with SLBO, in contrast to all the above-presented nitride and oxo-nitride red phosphors. All these structural characteristics and their influence on the emission properties of the study set of chosen phosphors will be thoroughly investigated below.

### Experimental Spectra

**Figure 2:**
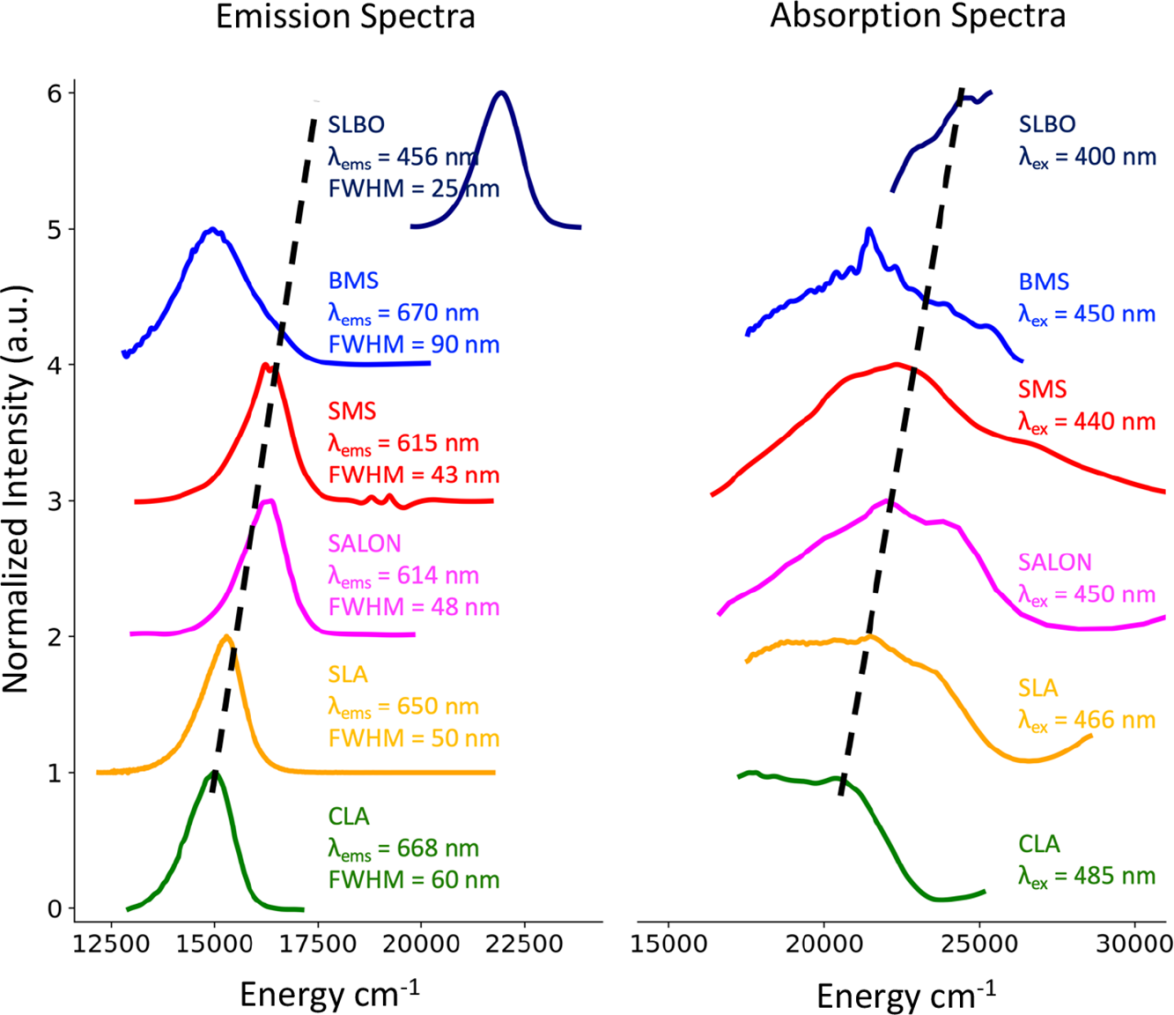
Experimental absorption (right) and emission (left) spectra of the selected phosphors (CLA: green; SLA: orange; SALON: pink; SMS: red; BMS: blue; SLBO: dark blue)

The experimental absorption and emission spectra of the chosen study set of Eu^2+^-doped phosphors are presented in Figure 2. As seen, all the absorption spectra are quite broad in the visible energy region 15,000–25,000 cm^–1^. The nitride phosphors CLA, SLA, and SMS show a broad absorption band with increasing band maximum positions observed at 21,280, 21,460, and 22,730 cm^–1^, respectively. In accordance, the oxo-nitride SALON and oxide SLBO phosphors show absorption spectra with band maxima at 22,220 and 23,100 cm^–1^, respectively. Interestingly, the band maxima increase in the sequence CLA, SLA, SALON ∼ SMS, SLBO. As will be discussed in the electronic structure section, this blue shift in the absorption maxima along the above sequence is associated with the subsequent decrease of the crystal field strength of the coordination environment around the Eu. On the contrary, BMS seems to deviate from this trend, showing a red shift in the absorption spectrum in which the band maximum position is located at 21,310 cm^–1^. In all cases, in the energy region 20,600–25,000 cm^–1^ (or wavelength region 400–485 nm), a single band emission line is observed in the energy region 12,500 to 22,500 cm^–1^ with a full width half-maximum (FWHM) that ranges between 1220 and 1990 cm^–1^ (25 and 90 nm). In particular, CLA, SLA, SALON, SMS, and SLBO show a narrow band emission with bandwidths ranging 60 nm (1330 cm^–1^) in CLA dropping down to 25 nm (1220 cm^–1^) in SLBO. According to the absorption spectra, the band maximum of the emission band is increasing along the sequence CLA (14,970 cm^–1^), SLA (15,385 cm^–1^), SALON (16,287 cm^–1^) ∼ SMS (16,260 cm^–1^), SLBO (21,930 cm^–1^) with the latter emitting quite deeply into the blue visible light frequency range (450–490 nm). In the case of the absorption spectrum of BMS, the respective emission spectrum declines from the above trend, showing a band maximum located at 14,925 cm^–1^, with a noticeably broad bandwidth (FWHM = 90 nm or 1990 cm^–1^). In the following sections, the intensity mechanism dominating the observed trends will be thoroughly investigated.

### Computational Details

All calculations were performed with the ORCA 5.0 suite of programs.^43−45^ Single-point energies and frequencies over the crystallographic coordinates, taken from the experimental crystallographic X-ray diffraction data, were computed at the density functional theory (DFT) level employing the PBE0^46,47^ functional. In all calculations, the def2-TZVP basis sets of the Ahlrichs group^48,49^ were used for all main group element atoms, while for Eu, the segmented all-electron relativistically re-contracted (SARC) scheme^50−53^ was employed. The calculations were accelerated by employing the resolution of identity approximation (RI)^54^ for the Coulomb integrals, while the exchange terms were efficiently computed using the “chain-of-spheres” (COSX)^55,56^ approximation by utilizing the def2/J and the SARC/J Coulomb fitting and def2-TZVP/C correlation auxiliary basis sets, respectively. All the calculations were performed using the second-order Douglas–Kroll–Hess correction (DKH2)^57,58^ to account for the scalar relativistic effects, employing the finite nucleus model.^59^ The Hartree–Fock (HF) layers used in the embedding cluster calculations were equipped with a minimal LANL2DZ basis set with the respective ECPs.^60−63^ Eu^2+^ doping energies were computed at the DLPNO-CCSD(T) levels of theory.^64−66^ Band gap energies of the host structures were computed at the TD-DFT(PBE0) and STEOM-DLPNO-CCSD^67,68^ levels of theory. In the TD-DFT calculations, the Tamm–Dancoff approximation (TDA) was used.^69^ Absorption and photoluminescence spectra were computed at the TD-DFT(PBE0)/TDA level of theory by employing the excited state dynamics (ESD) path integral protocol^70−72^ in which vibronic coupling is included within the Frank–Condon and Herzberg–Teller coupling schemes. A constant Gaussian broadening was used for all presented absorption and emission spectra, which amounts to a FWHM of 1500 and 500 cm^–1^, respectively. For better visual agreement with the experimental absorption spectra, a second Gaussian broadening with FWHM of 3000 cm^–1^ was used in some of the computed absorption spectra. For clarity, natural transition orbital (NTO) analysis is performed on dimer structures.

### Embedding Cluster Approach

#### Construction of the Cluster Models

**Figure 3:**
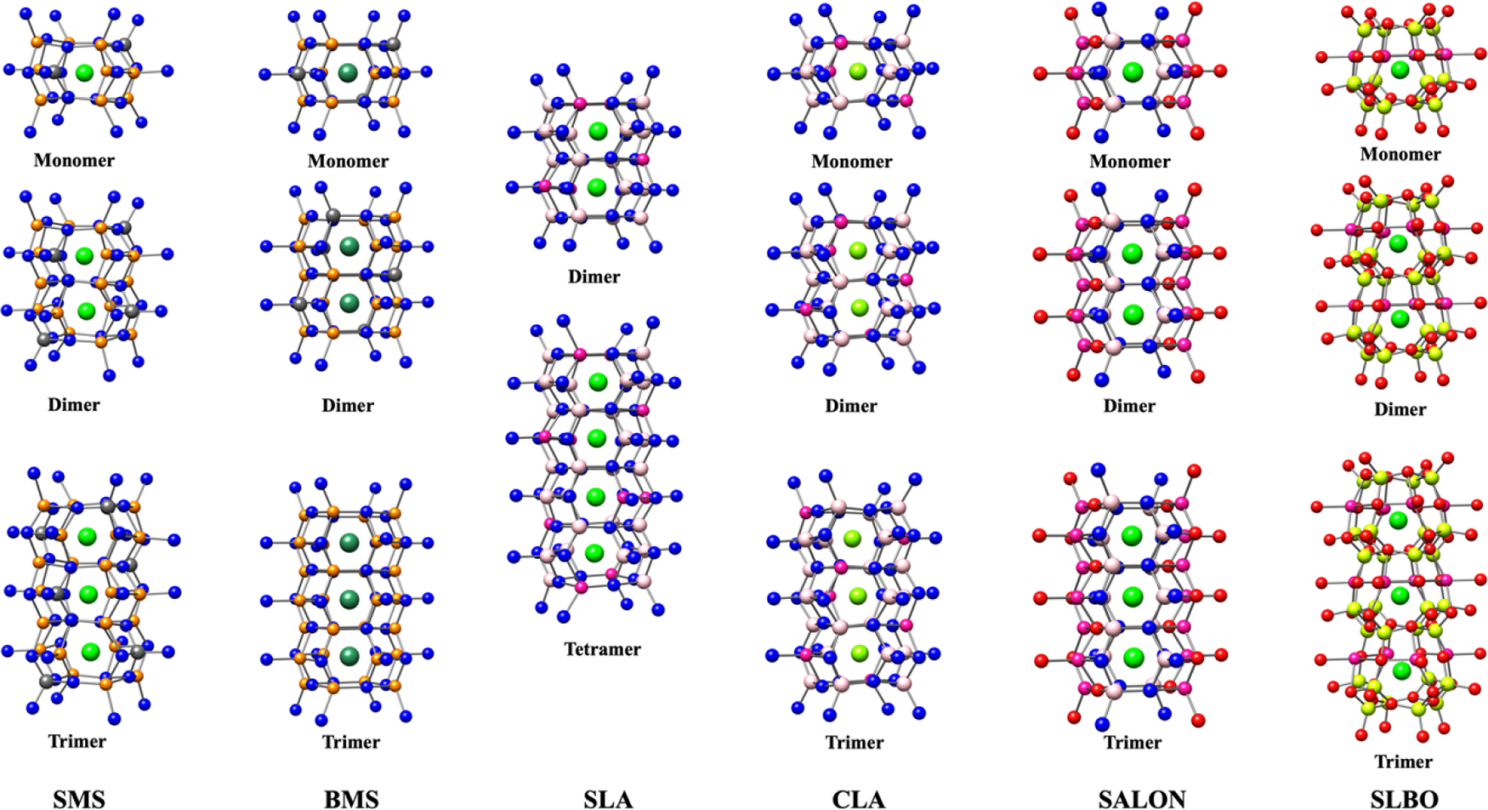
Graphical representation of (monomer, dimer, trimer, or tetramer) undoped clusters of SMS, BMS, CLA, SALON, and SLBO used for BG calculations. The respective doped clusters were constructed by replacing one of the central cations M (M = Ca^2+^, Sr^2+^, Ba^2+^), with Eu^2+^. Atom color coding: Ca (light green), Sr (green), Ba (deep green), Si (gray), Mg (orange), Al (yellowish pink), Li (pink), N (blue), O (red).

Representative model structures for the calculations of the spectroscopic properties of the host and Eu^2+^-doped phosphors were constructed in the framework of the embedded cluster approach. In a first step, various quantum clusters (QCs) were constructed from the crystallographic data by preserving the 8-fold cubic coordination environment around the central alkaline earth metal ions (Ca^2+^, Sr^2+^, Ba^2+^). Structure expansions containing one, two, three, or four central cations of the host phosphors were considered, abbreviated as monomers, dimers, trimers, or tetramers. The respective quantum clusters of the host structures of SMS, BMS, SLA, CLA, SALON, and SLBO phosphors are visualized in Figure 3.

**Figure 4:**
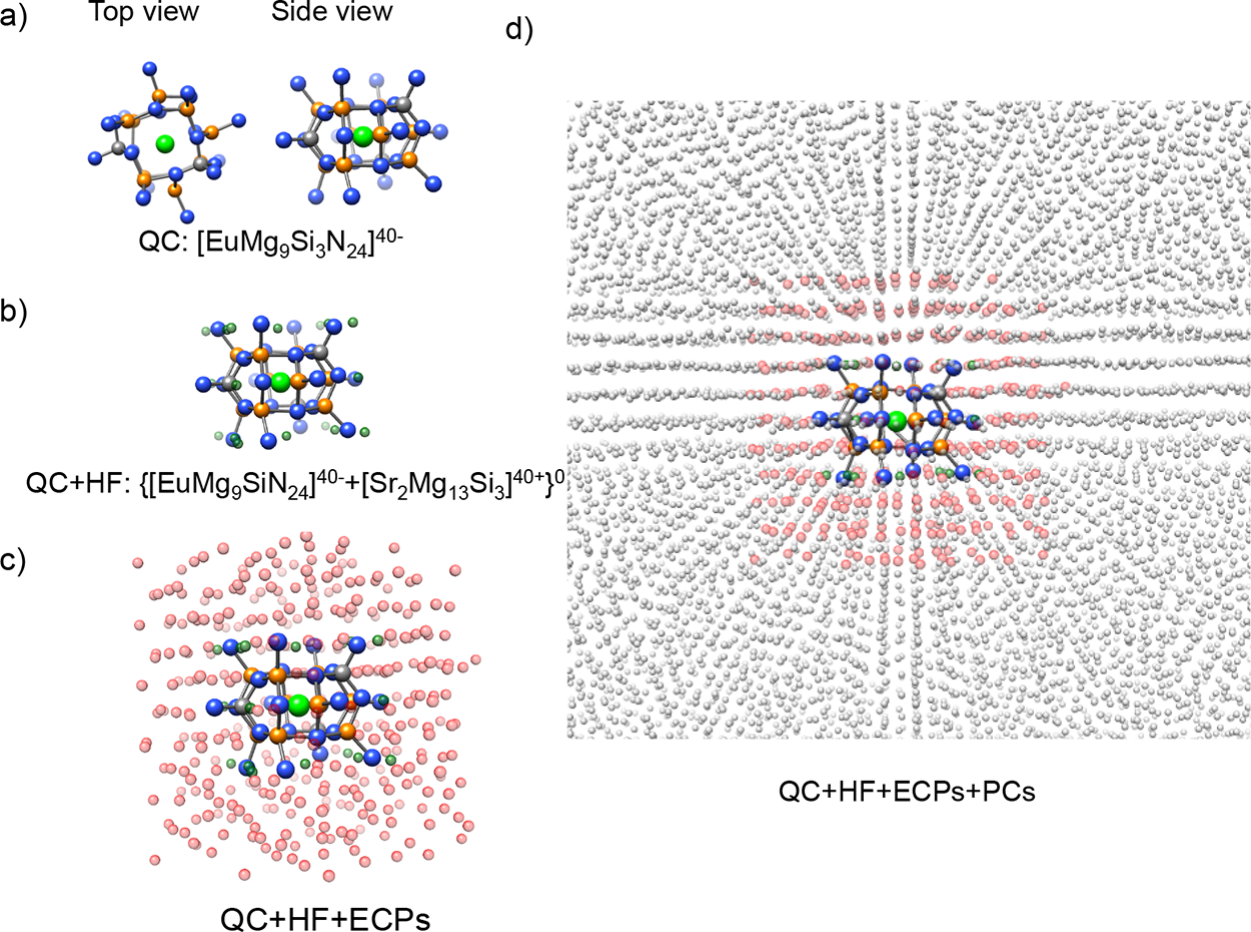
Embedding cluster approach. (a) Top and side views of the of the Eu^2+^-doped SMS [EuMg_9_SiN_24_]^48–^ QC cluster. (b) QC cluster surrounded by an HF layer (green spheres) composed of 2 Sr atoms, 13 Mg atoms, and 3 Si atoms playing the role of neutralizing the QC cluster QC + HF: {[EuMg_9_SiN_24_]^40–^ + [Sr_2_Mg_13_Si_3_]^40+^}^0^. (c) QC + HF cluster surrounded by three capped ECP layers (red spheres). (d) Final embedded cluster QC + HF + ECPs embedded in a PC (gray cycles) field. Atom color coding: Ca (light green), N (blue), Mg (orange), and Si (light gray).

Formation of Eu^2+^-doped phosphors involves the substitution of the Sr^2+^, Ca^2+^, or Ba^2+^ cations with Eu^2+^ due to the similarity of their ionic radii [Eu^2+^: 1.25, Ca^2+^: 1.06, Sr^2+^: 1.26, Ba^2+^: 1.35 Å].^73^ It should be noted that in this study set of phosphors, no other cation substitutions (e.g., Li^+^, Mg^2+^, Al^3+^, or Si^4+^) with Eu^2+^ are possible while occupation of interstitial sites on the unsaturated channels is not possible due to steric reasons.^42^ For example, in the case of SMS, the EuN_8_ host cuboid has a volume of 37 Å^3^ while the nearby unsaturated channels would form EuN_6_ octahedra with a 20 Å^3^ volume. Preliminary DLPNO-CCSD(T) calculations indicate that such Eu–polyhedra formations do not lead to more stable structures with respect to the Eu^2+^-doped cuboids as they require significant structural relaxation. As shown in Table S2, similar observations are found for all the phosphors of the study set. Hence, the corresponding candidate structures for the respective Eu^2+^-doped phosphors are straightforwardly constructed from the host model structures of Figure 3 by replacing one Sr^2+^, Ca^2+^, or Ba^2+^ cation with Eu^2+^. As shown in Table S1, all these clusters are highly negatively charged; hence, in a next step, one layer equipped with the surrounding cations is added. As described in the Computational Details, this layer, also called a Hartree–Fock layer (HF layer), is treated at the HF level during the calculation together with a minimal basis set. In following, the QC + HF clusters are embedded in an external point charge (PC) field, consisting of about 35,000 to 45,000 charges, to account for the long-range Coulombic forces. In order to avoid electron flow and overdelocalization from the QCs to the PC region, a boundary region (BR), 2 or 3 layers, of repulsive capped effective core potentials (c-ECPs) is introduced between the QC and PC regions. This region is also called an ECP region. In particular, an up to triple layer of cECPs:ECP2SDF (Li, Be, N),^74^ ECP10SDF (Sr, Mg, Ca, Si, Al)^75^ (included in the SDD framework) was used to replace the corresponding atoms. For the cECPs and PCs, the charges are chosen imposing cluster neutrality conditions (i.e., q(QC + HF) = – q(BR + PC))^76^ and by ensuring uniform charge distribution in the QC, BR, and PC regions. For this purpose, the charge values of the ECP and PC regions were matched with the computed electrostatic potential charges (CHELPG)^77,78^ of the QC iteratively. Further details regarding the employed embedding scheme have been described elsewhere.^79^ It should be noted that within the employed embedding scheme, the positions and magnitudes of the point charges are kept fixed while no additional corrections for the long-range electrostatics are taken into account.^80^ An example is provided in Figure 4 for the monomeric SMS {[EuMg_9_Si_3_N_24_]^40–^ [Sr_2_Mg_13_Si_3_]^40+^}^0^ cluster. This scheme has been proven successful in treating a variety of chemical problems in the field of semiconductors and insulators as well as molecular crystals.^79,81−90^ Note that while this scheme can be applied to a broad range of systems, metallic systems or materials for which the electronic structure is strongly delocalized cannot be treated.

#### Cluster Size Convergence

**Figure 5:**
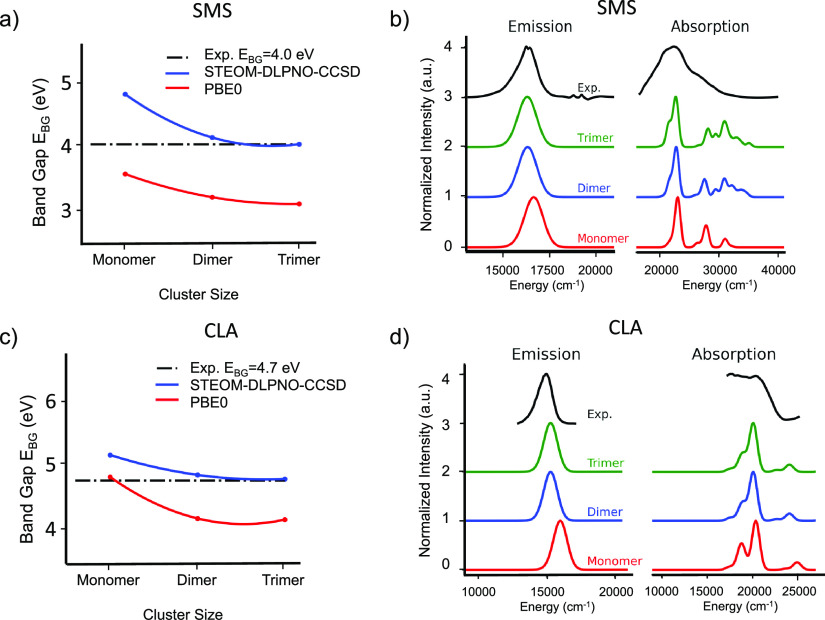
Cluster size convergence of SMS and CLA phosphors with respect to optical band gap energies and (a, c) absorption and fluorescence spectra (b, d). Band gap energies are computed at the PBE0 TD-DFT and STEOM-DLPNO-CCSD levels of theory while the absorption and fluorescence spectra at the PBE0 TD-DFT level in the framework of ESD approach are used.

In a next step, we perform cluster size convergence of the candidate clusters presented in Figure 3 and Table S1 with respect to the optical band gap (BG) energies of the host phosphor structures and the absorption and emission spectral shapes of the Eu^2+^-doped phosphors. For these purpose, we choose to present the cases of SMS^16^ and CLA.^12^ As seen in Figure 5a,c, the computed optical band gap energies at the PBE0 TD-DFT and STEOM-DLPNO-CCSD levels of theory in both cases are converged for the trimeric structures ({[EuSr_2_Mg_21_Si_7_N_48_]^68–^ + [Sr_2_Mg_22_Si_5_]^68+^}^0^ and {[EuCa_2_Li_7_Al_21_N_48_]^68–^ + [Ca_2_Li_7_Al_19_]^68+^}^0^). However, only when the STEOM-DLPNO-CCSD method is employed, the computed BG energies of the SMS and CLA host trimer structures are matching the experimental values with errors that are below 0.05 eV, while the respective PBE0 TD-DFT results deviate more than 0.5 eV from the experimental values. Nevertheless, this is still an acceptable deviation showing that PBE0 TD-DFT is a valid method to describe the absorption and emission spectra of these systems. In fact, as shown in Figure 5b,d, the shape of the PBE0 TD-DFT computed absorption and emission spectra of the Eu^2+^-doped trimer structures has converged while both types of spectra show nice agreement with the experiment. It should be emphasized that at the converged cluster sizes (e.g., trimer structures), placing Eu^2+^ at the center or the edge cuboids does not alter the computed quantities. Hence, based on the above results, in the case of SMS, BMS, CLA, SALON, and SLBO, the trimer Eu^2+^-doped structures have been chosen to study the absorption and fluorescence spectra of the study set of Eu^2+^-doped phosphors. In the case of SLA, tetramer clusters were used due to the two possible doping sites.

### Absorption and Emission Processes: A Qualitative Electronic Structure Analysis

Let us now discuss the most important factors that influence the absorption and emission processes in Eu^2+^-doped phosphors in which Eu^2+^ is doped in 8-fold coordinate host environments. For this purpose, we will undertake an electronic structure analysis based on the monomer (dimer, trimer, etc.) structures presented in Figure 3.

**Scheme 1:**
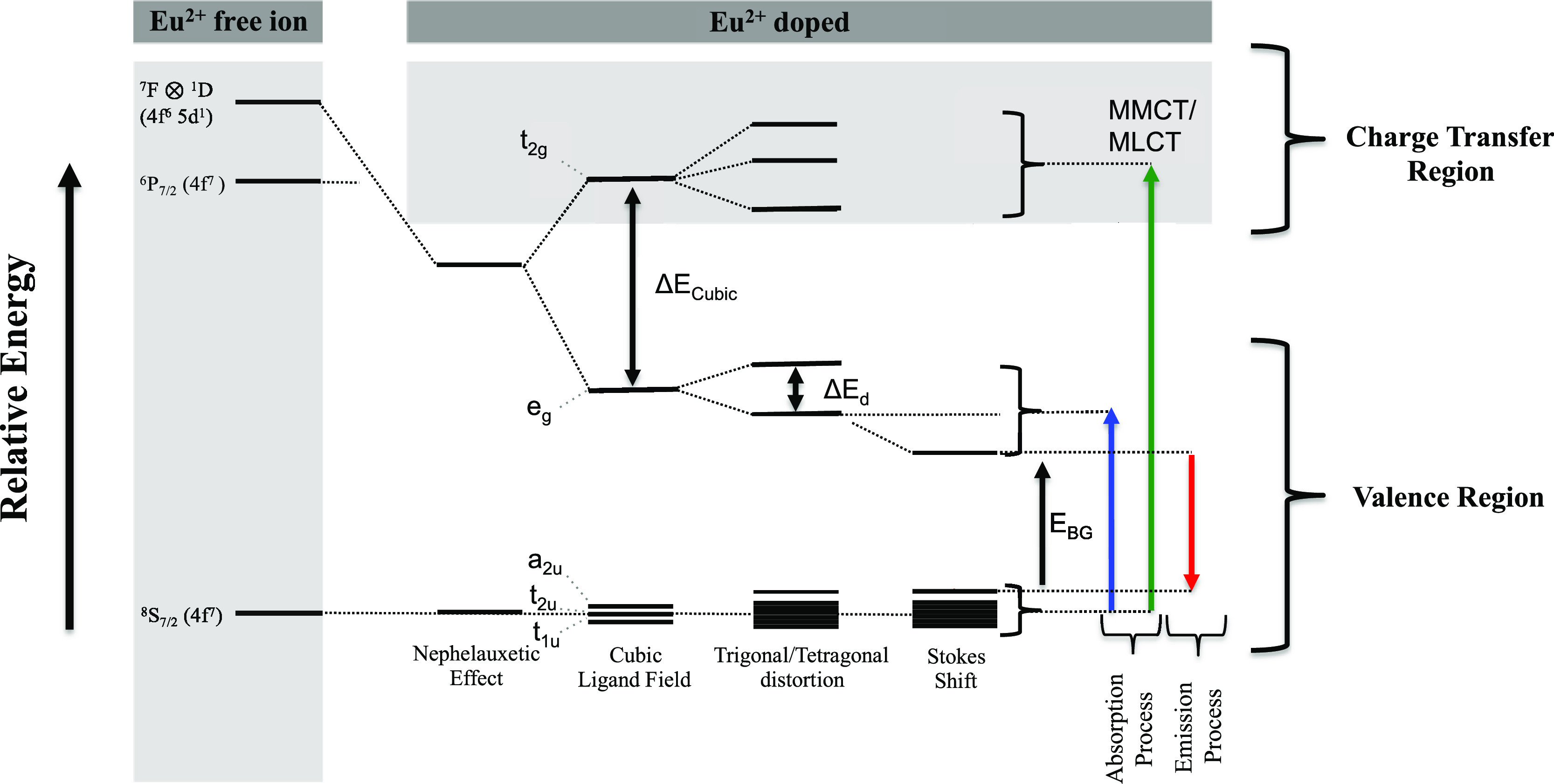
Schematic Representation of the Absorption and Emission Processes in Eu^2+^-Doped Phosphors Together with the Most Important Energy Quantities That Dominate the Energy Position and the Bandwidth of the Spectral Features

The divalent europium ion (Eu^2+^) has a [Xe]4f^7^ electronic configuration with a half-filled f^7^ orbital. The f shell is highly shielded due to the closed shell 5s^2^ and 5p^6^ outer shells. The seven electrons in the f shell can be arranged in many different ways leading to 3432 degenerate atomic microstates (2 octet (S = 7/2), 98 sextet (S = 5/2), 882 quartet (S = 3/2), and 2450 doublet (S = 1/2)). This degeneracy can be partly or totally lifted due to several perturbations like interelectronic repulsion, ligand field splitting, spin orbit coupling, or even the Zeeman effect. In the ion, the half-filled f shell leads to a very stable octet ground state (^8^S_7/2_) multiplet with a 4f^7^5d^0^ electron configuration of multiplicity 2S + 1 = 8 (where S is the total spin). The lowest excited state atomic multiplet arises by a spin-flip type of excitations of the same 4f^7^5d^0^ electron configuration with multiplicity 2S + 1 = 6 (^6^P_7/2_), which is difficult to reach as it is separated by >4.5 eV from ^8^S_7/2_.^91^ The other and more interesting excitation pathway is to excite electron(s) to the empty 5d orbitals via spin-conserving 4f → 5d single electron excitations involving the 4f^6^5d^1^ electron configurations with multiplicity 2S + 1 = 8 (^7^F ⊗ ^1^D). In the ion, this excited shell remains still high in energy. As shown in Scheme 1, when Eu^2+^ is doped in 8-fold coordinated host environments, under the 1-electron picture, the action of interelectronic repulsion will stabilize the 2S + 1 = 8 excited state multiplets of the 4f^6^5d^1^ shell above the 2S + 1 = 8 4f^7^5d^0^ ground state multiplets. It follows that the cubic ligand field splitting will lift the degeneracy of the 4f and 5d orbitals in an inverted octahedral order (Δ_cubic_ = −8/9Δ_Oh_) leading to a ground state electron configuration t_1u_^3^ t_2u_^3^ a_2u_^1^ e_g_^0^ t_2g_^0^. Further distortions toward tetragonal/trigonal ligand fields will further lift the remaining orbital degeneracies and consequently the ground- and excited-state degeneracy. It follows that quantities like ligand field splittings (Δ*E*_Cubic_, Δ*E_d_*), band gap energies, *E*_BG_, and Stokes shifts are important quantities of the absorption and emission processes, which determine the energy position and the bandwidth of the different spectral features. In addition, as will be explicitly discussed, the energy difference between the valence Eu 4f → Eu 5d_*z*2/*x*2 – *y*2_ and the metal to metal charge transfer Eu 4f → Eu 5d_*xz*/*yz*/*xy*_ (MMCT) excitations or the metal to ligand charge transfer Eu 4f → L 3p_*x*/*y*/*z*_ (MLCT) can be thought of as a measure of the Eu^2+^-doped phosphors’ thermal stability.^21^ In fact, it has been shown that synchrotron-based X-ray techniques like nitrogen 1s2p resonance X-ray emission (RXES) spectroscopy provide a direct measurement of this energy separation in nitride phosphors, making it a key indicator of quantum efficiency.^21^

**Figure 6:**
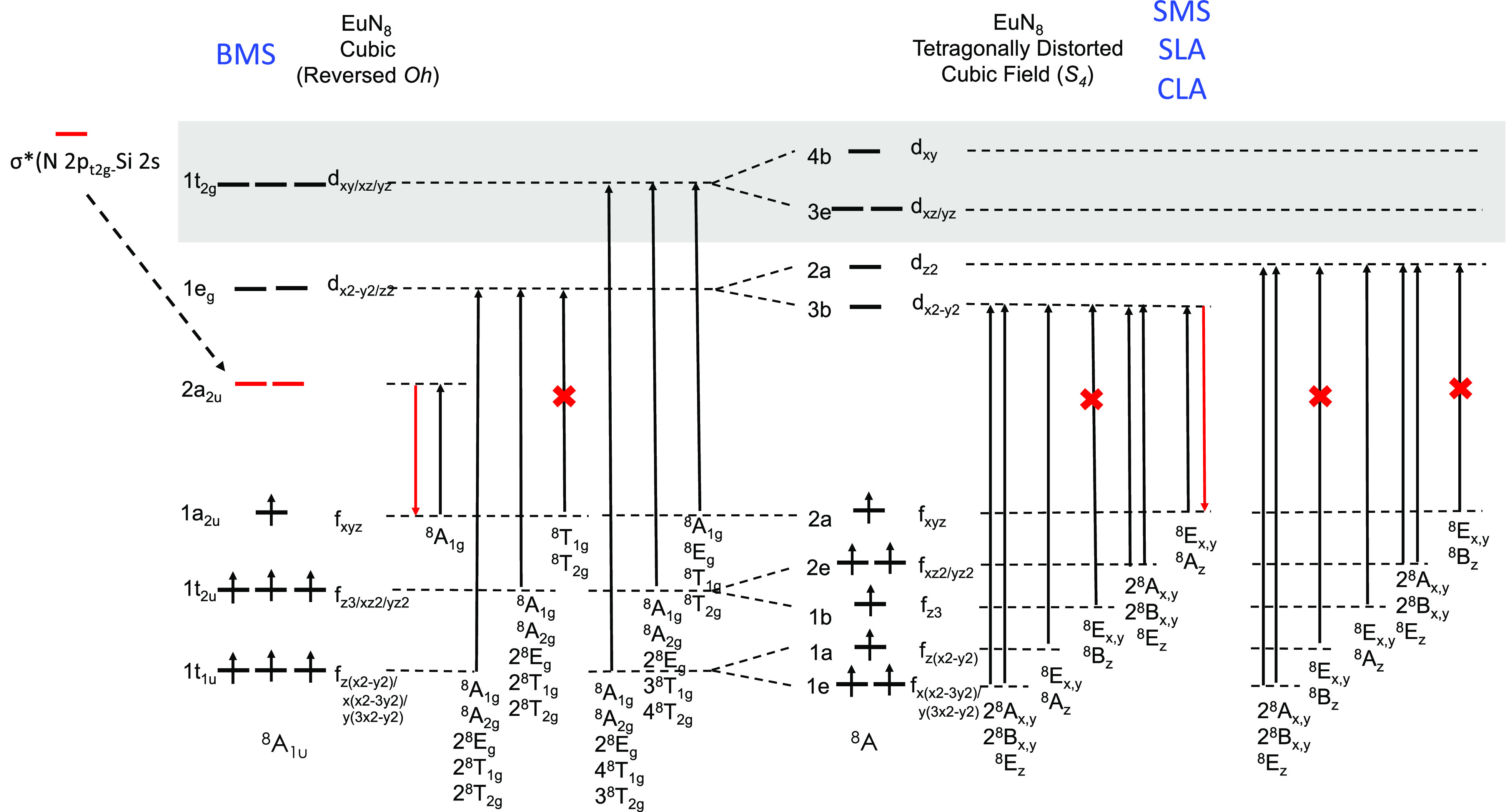
Qualitative molecular orbital diagram of the monomeric of nitride doped Eu^2+^ phosphors adopted for cubic (reversed *O_h_*) EuN_8_ centers (BMS) and for EuN_8_ centers (SMS, SLA and CLA) of in *S*_4_ symmetry. Black arrows indicate absorption processes. Red arrows indicate emission processes and red crosses indicate dipole forbidden transitions.

Focusing now on the Eu^2+^-doped nitride phosphors (SMS, BMS, SLA and CLA), while in BMS, Eu^2+^ is coordinated in an 8-fold N^3–^ network in an approximate cubic symmetry, in SMS, SLA, and CLA, a tetragonal distortion occurs around the Eu^2+^ center, which reduces the symmetry of the EuN_8_ building block from cubic (inverted *Oh*) to *D*_4*h*_. In reality, due to additional distortions in the host frame, the center of inversion is lost reducing further the symmetry of EuN_8_ to *S*_4_. The molecular orbital (MO) energy splitting of the ground-state configurations of BMS, SMS, SLA, and CLA, in cubic and *S*_4_ symmetries, together with the most important single electron excitations arising from these ground state electron configurations within the 1-electron picture is visualized in Figure 6. In SMS, SLA, and CLA, the *S*_4_ symmetric ^8^A ground state obtains the 1e^1^1a^1^1b^1^2e^1^2a^1^3b^0^2a^0^3e^0^4b^0^ electron configuration. It follows that valence Eu 4f → Eu 5d_*z*2/*x*2 – *y*2_ single electron excitations or electron decays Eu 5d_*z*2/*x*2 – *y*2_ → Eu 4f will give rise to 2S + 1 = 8 and 2S + 1 = 6 multiplets of A and E symmetries along the absorption and emission processes, respectively. BMS on the contrary obtains an ^8^A_1u_ ground state. As illustrated in Figure 6 under cubic symmetry, the Si-2s and N-2p orbitals in the SiN_4_ cuboid are correctly oriented to allow maximum overlap. As a result, the ligand-based antibonding σ*(Si-2s, N-2p) MO is stabilized below the Eu-5d MOs leading to the ground-state 1t_1u_^3^ 1t_2u_^3^ 1a_2u_^1^ 2a_2u_^0^ 1e_g_^0^ 1t_2g_^0^ electron configuration. Hence, the lowest multiplets in the absorption and emission processes will be dominated by Eu 4f_xyz_ → L σ*(Si – 2s, N – 2p) and L σ*(Si – 2s, N – 2p) → Eu 4f_xyz_ electron excitations and electron decays, respectively.

**Figure 7:**
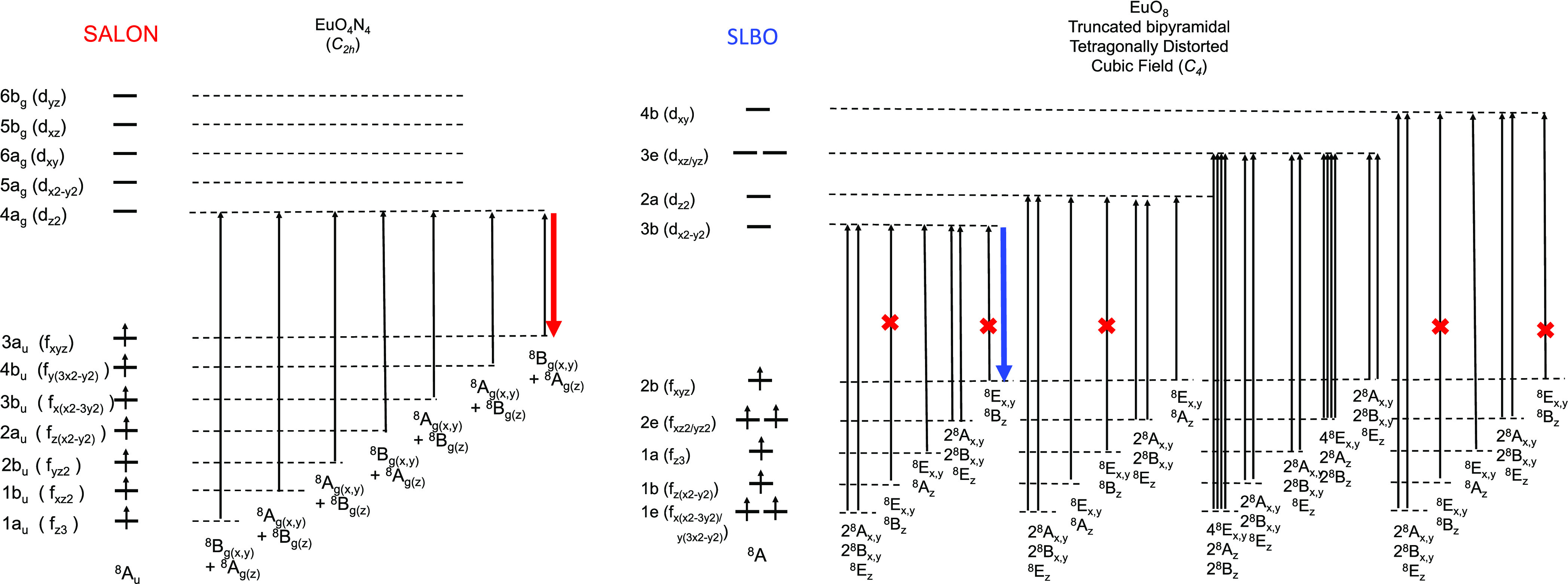
Qualitative molecular orbital diagram of the monomeric oxo-nitride and oxide-doped Eu^2+^ phosphors adopted for cubic (reversed *O_h_*) EuO_4_N_4_ centers (SALON) and for tetragonally distroted (C_4_) EuO_8_ centers (SLBO). Black arrows indicate absorption processes. Red arrows indicate emission processes and red crosses indicate dipole-forbidden transitions.

Similar to Eu^2+^-doped nitride phosphors SMS and SLA, oxo-nitride SALON and oxide SLBO phosphors show distorted cubic geometries in which the EuO_4_N_4_ and EuO_8_ building units show *C*_2*h*_ and *C*_4_ symmetries around the Eu^2+^ centers (Figure 7). Under these symmetry assumptions, SALON has an ^8^A_u_ ground state with a 1a_u_^1^1b_u_^1^2b_u_^1^2a_u_^1^3b_u_^1^4b_u_^1^3a_u_^1^4a_u_^0^5a_u_^0^6a_u_^0^5b_u_^0^6b_u_^0^ electron configuration while SLBO has an ^8^A ground state with a 1e^2^1b^1^1a^1^2e^2^2b^1^3b^0^2a^0^3e^0^4b^0^ electron configuration. In both cases, valence Eu 4f → Eu 5d_*z*2/*x*2 – *y*2_ single electron excitations or electron decays Eu 5d_*z*2/*x*2 – *y*2_ → Eu 4f will give rise to 2S + 1 = 8 and 2S + 1 = 6 multiplets of A and B symmetries along the absorption and emission processes, respectively.

## Results and Discussion

### Theoretical Protocol for Understanding the Absorption and Fluorescence Spectra as well as the Thermal Stability of Narrow Band Phosphors

#### Nitride Phosphors

All the above qualitative observations will be used in this section in order to develop a theoretical protocol that is able to (1) predict the absorption and fluorescence spectra band shapes, (2) provide insight on the nature of the dominating spectral features, and (3) predict the thermal stability of the different phosphors. For this purpose, we first focus on the nitride Eu^2+^-doped phosphors SMS, BMS, SLA, and CLA. As discussed in the computational section in more detail, the absorption and fluorescence spectra are computed at the PBE0 TD-DFT level of theory in the framework of the ESD approach. Analysis of the spectral features is performed on the basis of natural transition orbitals (NTOs).

**Figure 8:**
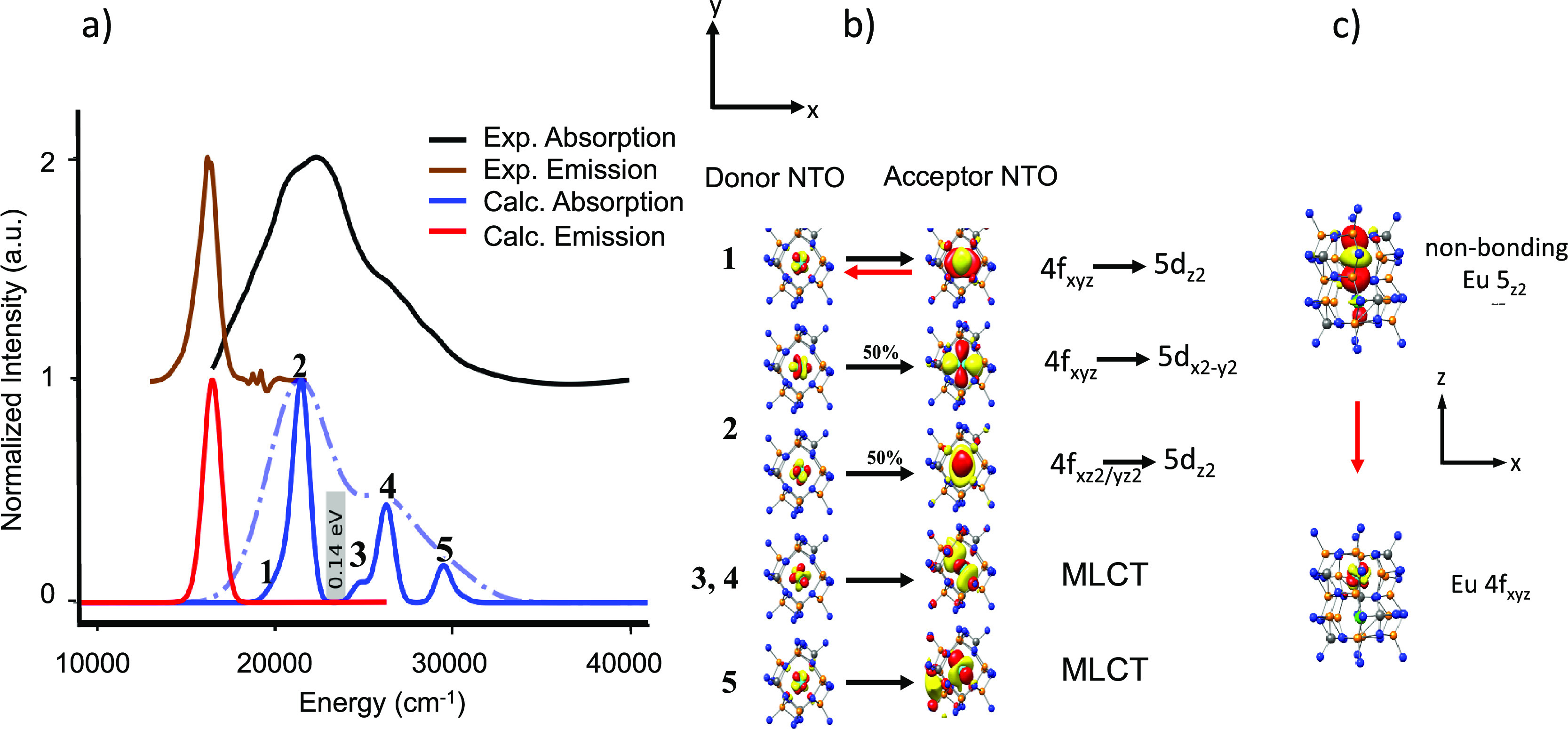
(a) SMS experimental (black), calculated TD-DFT/PBE0 absorption (blue, light blue) spectra and experimental (brown), and TD-DFT/PBE0/ESD-calculated (red) emission spectra. (b) NTO analysis of the relevant bands in absorption spectra and (c) the 1st transition responsible for emission upon relaxation.

Starting with SMS, the computed versus experimental absorption and fluorescence spectra are visualized in Figure 8. As seen, the agreement between theory and experiment is very good, allowing a quantitative analysis of the spectral features. In fact, four bands dominate the absorption spectrum. The first two bands (1 and 2) according to the NTO analysis involve Eu 4f_xyz_ → Eu 5d_*z*2_ Eu 4f_xyz_ → Eu 5d_*x*2 – *y*2_ and Eu 4f_xz2/yz2_ → Eu 5d_*x*2 – *y*2_ single electron excitations while the shoulder (band 3) and the other two bands (4 and 5) are dominated by an MLCT type of excitation. Band 1 shows that the emission process involves electron decay from a nonbonding Eu 5d_z2_ orbital to an isolated Eu 4f_xyz_ orbital. Since there is no significant distortion in the excited state relative to the electronic ground state, there can also not be any vibronic progression, thus leading to narrow band emission. Consequently, the rigid nature of this transition causes narrow band emission. The energy separation between the valence bands (1 and 2) and the MLCT bands (3–5) is ∼1150 cm^–1^ (0.14 eV). Such a small energy separation indicates that thermal quenching of the emission through non-radiative relaxation processes is possible explaining the poor thermal stability of SMS. In fact, the computed emission spectra from bands 3 and 4 lead to negligible intensity emission lines.

**Figure 9:**
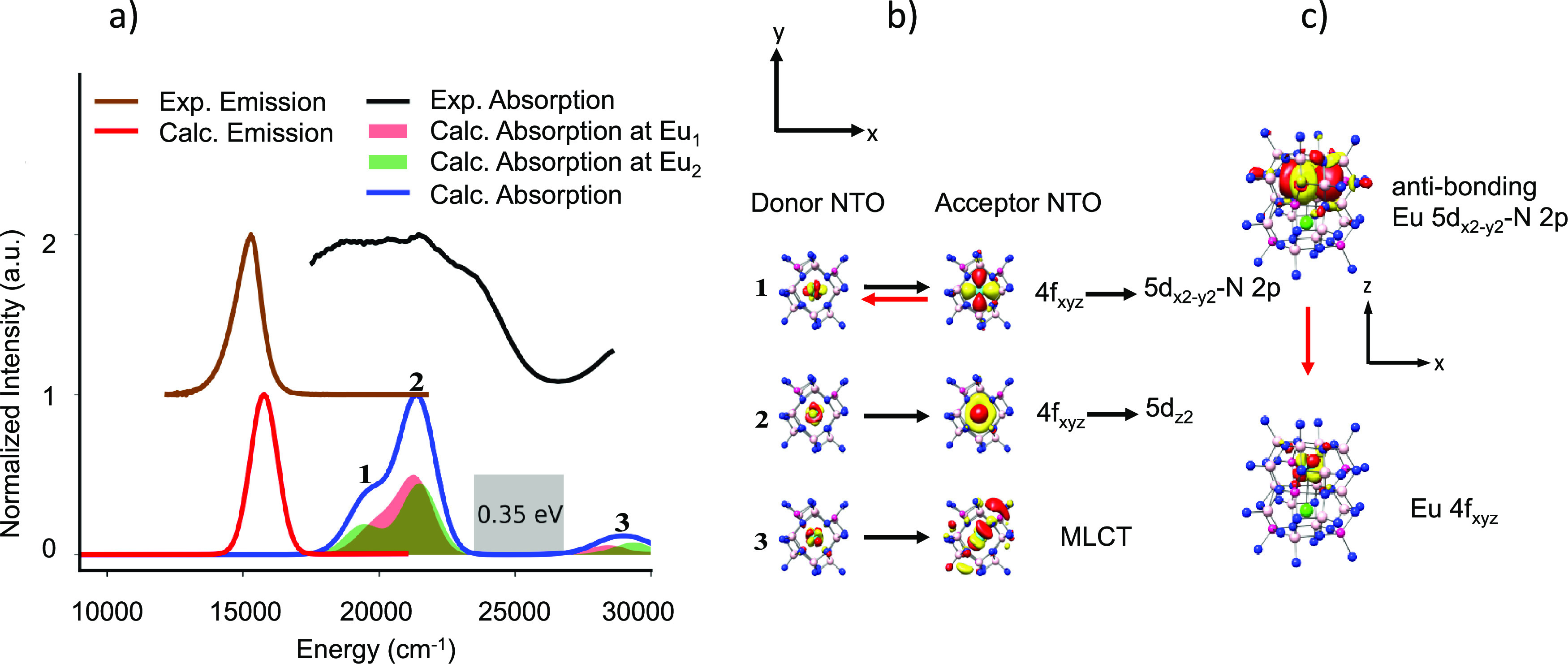
(a) SLA experimental (black), calculated TD-DFT/PBE0 absorption (blue, light blue) spectra and experimental (brown), and TD-DFT/PBE0/ESD-calculated (red) emission spectra. (b) NTO analysis of the relevant bands in absorption spectra and (c) the 1st transition responsible for emission upon relaxation.

Let us now discuss the case of the narrow band phosphors SLA and CLA. These phosphors have shown somewhat larger emission bandwidths (SLA_FWHM_ = 50 nm/1190 cm^–1^, CLA_FWHM_ = 60 nm/1330 cm^–1^) with respect to that of SMS (SMS_FWHM_ = 43 nm/1170 cm^–1^). The computed versus experimental absorption and fluorescence spectra are visualized in Figure 9 and Figure S1. Once again, the agreement between theory and experiment is satisfactory, allowing a quantitative analysis of the experimentally observed spectral features. In the case of SLA presented in Figure 9, there are two different Eu doping candidate sites, with similar local geometric and electronic structure. This also leads to similar absorption spectra and hence to similar emission spectra maintaining the narrow bandwidth. The computed absorption spectrum is dominated by three bands, which according to the NTO analysis are characterized by Eu 4f_xyz_ → Eu 5d_*x*2 – *y*2_ – N 2p (band 1) Eu 4f_xyz_ → Eu 5d_*x*2 – *y*2_ (band 2) and metal to ligand charge transfer (MLCT, band 3) single electron excitation contributions. As band 1 indicates, the emission process involves an electron decay from a practically antibonding Eu 5d_*x*2 – *y*2_ – N 2p molecular orbital to an isolated Eu 4f_xyz_ orbital. This introduces little vibronic interaction with the environment, which is associated with the 2% N 2p character of the acceptor NTO orbital that dominates absorption band 1. Once again, the rigid nature of this transition causes narrow band emission; however, due to the antibonding character of the acceptor NTO orbital participating in the emission process, the SLA emission spectra are broader than those of SMS. On the contrary, the higher thermal stability of the SLA phosphor in comparison to SMS is associated with the large energy separation between the valence and MLCT bands in the absorption spectrum, which amounts to 0.35 eV in SLA in comparison to 0.14 eV in SMS. This is also in agreement with experimental observation from direct nitrogen 1s2p RXES measurments.^21^

In the case of CLA, the computed absorption spectrum presented in Figure S1 shows five bands, which according to the NTO analysis are characterized by Eu 4f_xyz_ → Eu 5d_*x*2 – *y*2_ – N 2p (band 1) Eu 4f_xz2/yz2_ → Eu 5d_*x*2 – *y*2_ – N 2p (band 2), Eu 4f_xz2/yz2_ → Eu 5d_*z*2_, and metal to ligand charge transfer (MLCT, shoulder band 4 and bands 5 and 6) single electron excitation contributions. As in the case of SLA, band 1 indicates that the emission process involves an electron decay from a practically antibonding Eu 5d_*x*2 – *y*2_ – N 2p molecular orbital to an isolated Eu 4f_xyz_ orbital, which again introduces little vibronic interaction with the environment.

In comparison to SLA, the N 2p contribution to the NTO donor orbital dominating band 1 increases from 2 to 4%. Such an increase in the NTO orbital antibonding character is associated with the higher degree of compression of the EuN_8_ cuboid and consequently the further increase of the crystal field strength in CLA in comparison to SLA. As a result, a narrow band emission is observed with a larger bandwidth with respect to both SLA and SMS. The energy separation between the valence band (3) and MLCT (5) band is ∼1700 cm^–1^ (0.21 eV), indicating an intermediate thermal stability between SLA and SMS.

**Figure 10:**
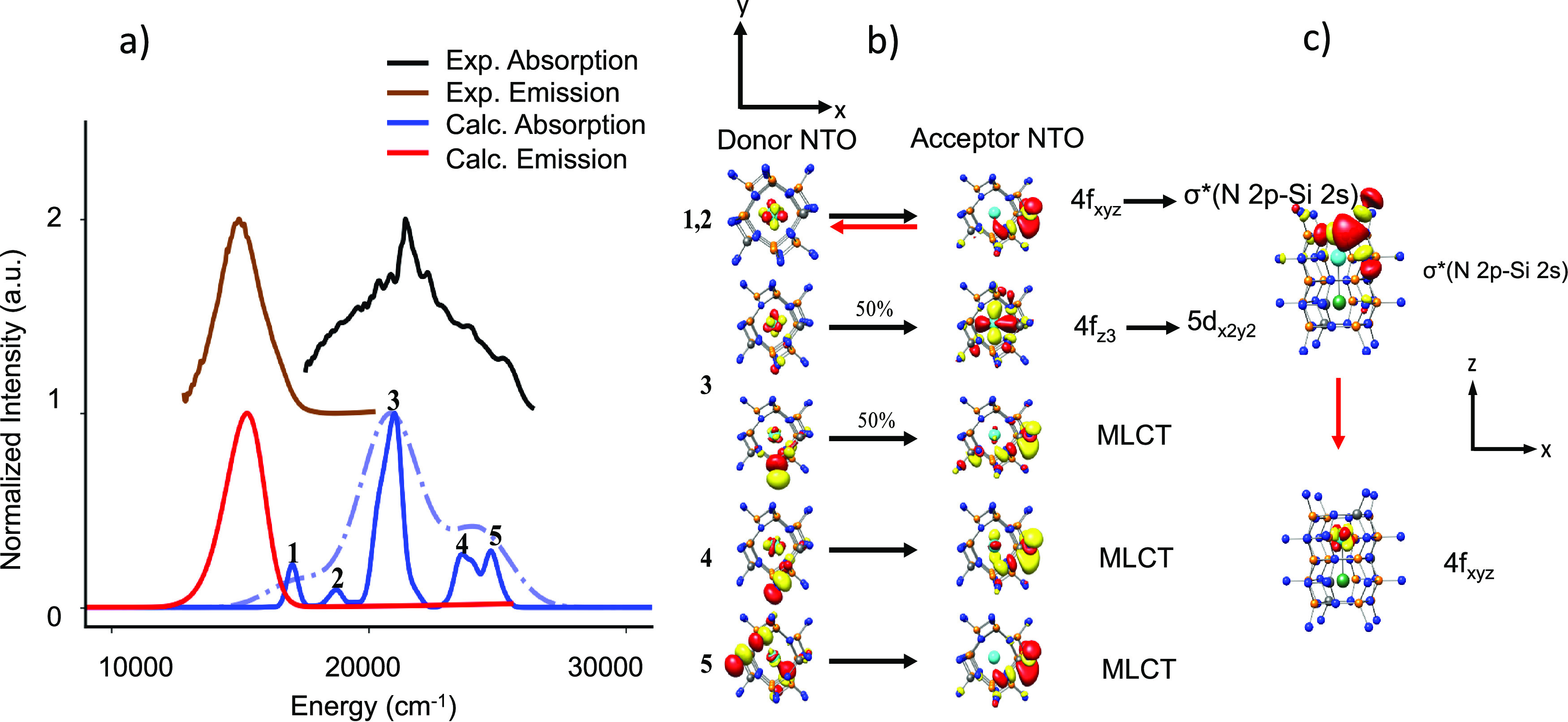
(a) BMS experimental (black), calculated TD-DFT/PBE0 absorption (blue, light blue) spectra and experimental (brown), and TD-DFT/PBE0/ESD-calculated (red) emission spectra. (b) NTO analysis of the relevant bands in absorption spectra and (c) the 1st transition responsible for emission upon relaxation.

In the case of BMS, the situation changes rapidly. As discussed in Figure 6, the ligand-based antibonding σ*(Si-2 s, N-2p) MO is stabilized below the Eu-5d MOs. This situation is reflected to both the absorption and emission spectra. The computed absorption spectrum presented in Figure 10 indicates that five bands dominate the broad unresolved experimental spectral feature in the energy range 18,000 and 26,000 cm^–1^. According to the NTO analysis, all these bands have a significant metal to ligand charge transfer character (MLCT). In particular, bands 1 and 2 in contrast to all other nitride phosphors have a pure MLCT character dominated by Eu 4f_xyz_ → σ*(N 2p – Si 2s) single electron excitation. Band 3 has mixed valence Eu 4f_z3_ → Eu 5d_*x*2 – *y*2_ – N 2p and MLCT characters while bands 4 and 5 have solely MLCT single electron excitation characters. Hence, the emission process from band 1 results in a red-shifted emission spectrum in contrast to what is expected by the crystal field strength (Figures 1 and 2). This is also reflected by the experimental and computed Stokes shifts presented in Table 1. As seen, while the Stokes shifts in the case of SMS, SLA, and CLS range between 500 and 1000 cm^–1^, in the case of BMS, they are larger than 2500 cm^–1^. The bandwidth of the computed emission spectrum in accordance to the experimental one is also increased (FWHM = 90 nm/1990 cm^–1^) in comparison to the observed and computed bandwidths in SMS, SLA, and CLA. This is due to the non-rigid nature of the transition, in which vibrations within the host ligand framework participate and apparently dominate the band broadening mechanism. This is also supported by the computed fluorescence rates and relaxation times presented in Table S4. As seen in the Frank–Condon approximation and upon applying Herzberg–Teller corrections, in the case of BMS, the computed fluorescence rates are >3 orders of magnitude smaller while the respective relaxation times are about 5 orders of magnitude larger in comparison to the other phosphors reflecting a different relaxation pathway. Nevertheless, these relaxation times at the fluorescence time frame are very small and cannot be safely used to define rigidity.

**Table 1:** Experimentally Observed and Calculated Stokes Shifts for the Study Set of Phosphors

phosphor	experimental Stokes shift Δ*S* (cm^–1^)	calculated Stokes shift Δ*S* (cm^–1^)
Ba[Mg_3_SiN_4_]:Eu^2+^ BMS	3500	2810
Sr[Mg_3_SiN_4_]:Eu^2+^ SMS	750	950
Ca[LiAl_3_N_4_]:Eu^2+^ CLA	1000	847
Sr[LiAl_3_N_4_]:Eu^2+^ SLA	800	840
Sr[Al_2_Li_2_O_2_N_2_]:Eu^2+^ SALON	1100	1150
SrLi_2_[Be_4_O_6_]:Eu^2+^ SLBO	980	750

It should be noted that one of the most commonly used quantity to define rigidity and thermal quenching is the Debye temperature.^92,93^ The Debye temperature although it is a proxy of rigidity is not always predictive of thermal quenching.^94,95^ A more valid quantity is the Huang–Rhys factor *S*, which is a measure of the strength of the electron–phonon coupling in the emission process.^8,96,97^ In fact, along the study set, the computed Huang–Rhys factors presented in Table S3 follow the trend of the energy separation of the MLCT band. In particular, small Huang–Rhys factors that are indicative of structural rigidity are associated with large MLCT band separation, reflecting a higher thermally stable phosphor.

To conclude this part, we have developed in this section a computational protocol that is able to relate the emission bandwidth of the nitride phosphors to the nature of the single electron decay that dominates the emission process from the first excited state that is reached in the absorption spectrum. In the next section, we will apply this protocol to characteristic examples from the oxo-nitride and the oxide families of phosphors, namely, SALON and SLBO.

#### Oxo-nitride and Oxide Phosphors

**Figure 11:**
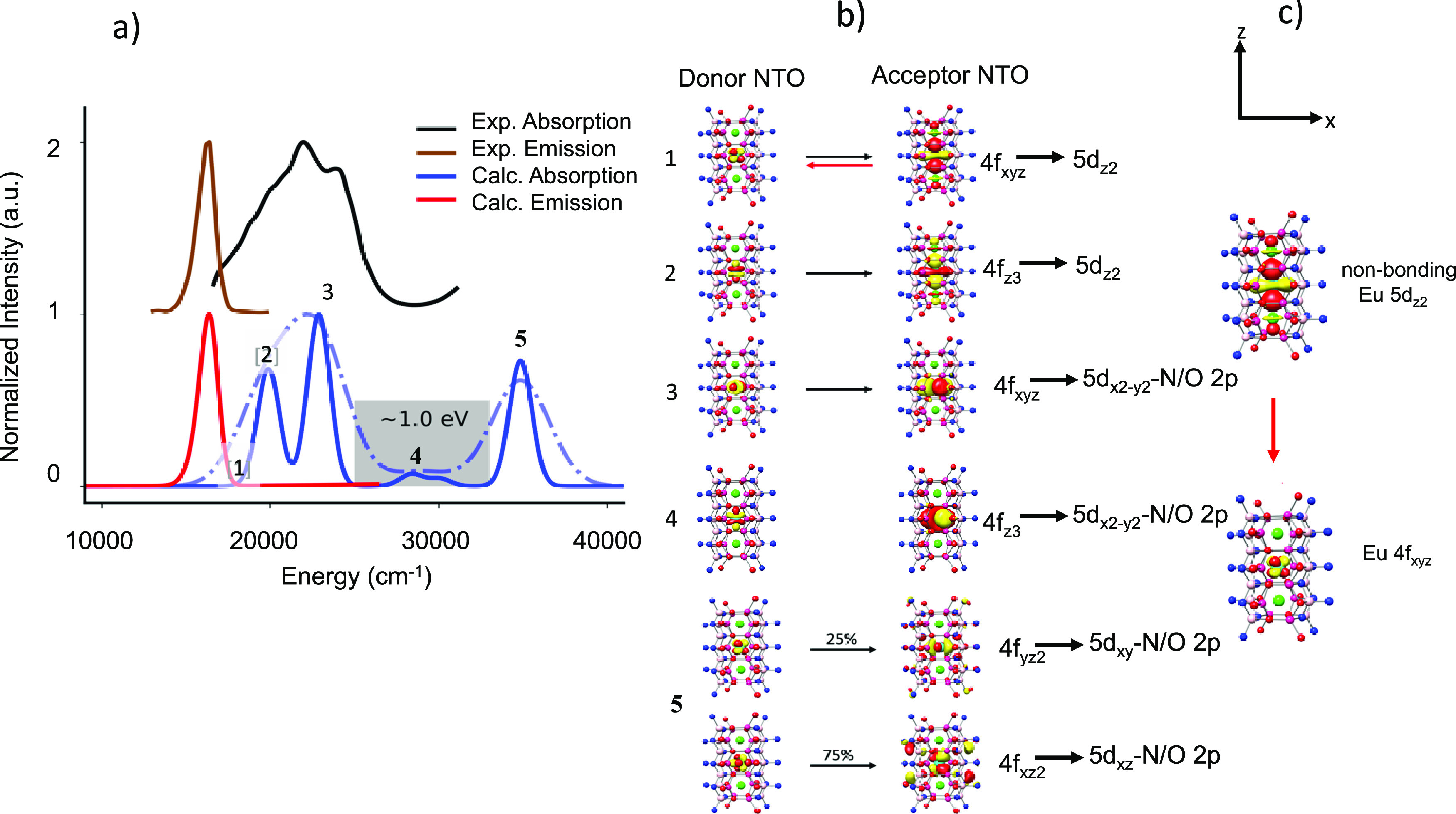
(a) SALON experimental (black), calculated TD-DFT/PBE0 absorption (blue, light blue) spectra and experimental (brown), and TD-DFT/PBE0/ESD-calculated (red) emission spectra. (b) NTO analysis of the relevant bands in absorption spectra and (c) the 1st transition responsible for emission upon relaxation.

In the case of SALON, the computed versus experimental absorption and fluorescence spectra are visualized in Figure 11. As seen, the agreement between theory and experiment is once again very good, thus allowing a quantitative analysis of the experimentally observed spectral features. The absorption spectrum consists of five bands, which according to NTO analysis all involve valence Eu 4f → Eu single electron excitations, namely, Eu 4f_xyz_ → Eu 5d_*z*2_ (band 1), Eu 4f_z3_ → Eu 5d_*z*2_ (band 2), Eu 4f_xyz_/4f_z3_ → Eu 5d_*x*2 – *y*2_ – N/O 2p (band 3, band 4), Eu 4f_yz2_ → Eu 5d_*xy*_ – N/O 2p, and Eu 4f_xz2_ → Eu 5d_*xz*_ – N/O 2p (band 5) single electron excitation contributions. According to SMS, the emission process involves an electron decay from a nonbonding Eu 5d_z2_ orbital to an isolated Eu 4f_xyz_ orbital, with practically negligible vibronic interaction with the lattice environment. Again, the rigid nature of the transition is the reason for the observed narrow bandwidth emission (46 nm /1220 cm^–1^). As described above, band 5 has a significant MLCT character. Hence, as in the case of SLA, the energy separation between the valence bands (1–4) and the MLCT band (5) (∼10,000 cm^–1^, ∼1.0 eV) is associated with an observed high thermal stability.

**Figure 12:**
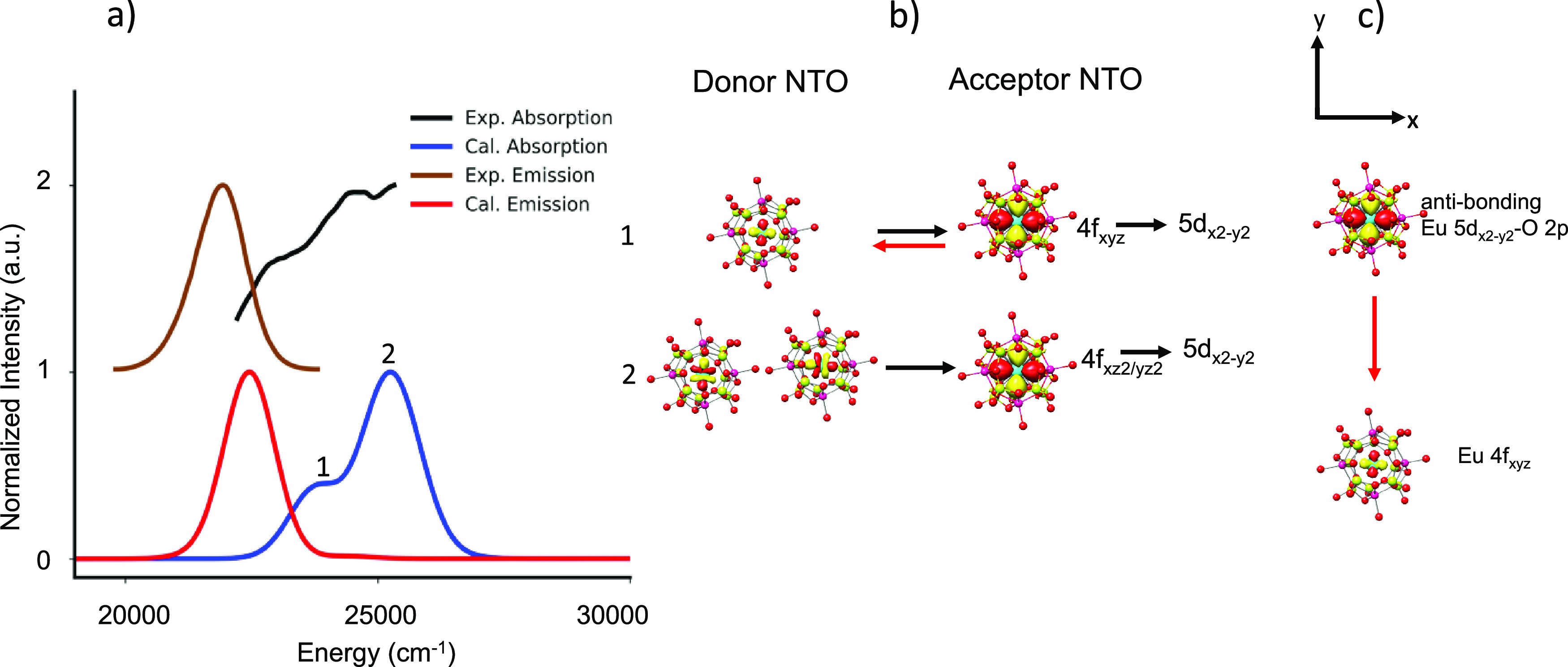
(a) SLBO experimental (black), calculated TD-DFT/PBE0 absorption (blue, light blue) spectra and experimental (brown), and TD-DFT/PBE0/ESD-calculated (red) emission spectra. (b) NTO analysis of the relevant bands in absorption spectra and (c) the 1st transition responsible for emission upon relaxation.

As a final example, we discuss the case of blue-emitting SLBO. The computed versus experimental absorption and fluorescence spectra are visualized in Figure 12. The good agreement between theory and experiment allows again for a quantitative analysis of the observed spectral features. According to NTO analysis, the absorption spectral features are dominated by the valence Eu 4f_xyz_ → Eu 5d_*x*2 – *y*2_ (band 1) and Eu 4f_xz2/yz2_ → Eu 5d_*x*2 – *y*2_ (band 2) single electron excitations. Under a *C*_4_ truncated square bipyramidal coordination environment of the EuO_8_ building units, the manifold of the f orbitals remains compact. This results in a blue shift of all the absorption bands in comparison to all other phosphors, which adopt distorted cubic EuN_8_ or EuN_4_O_4_ building units. In addition, in such a coordination environment around the Eu^2+^ center, the Eu 5d_x2 – y2_ MO remains essentially nonbonded. Once again, the rigid nature of the Eu 5d_*x*2 – *y*2_ → 4f_xyz_ transition is the reason for the observed narrow bandwidth emission (25 nm/1220 cm^–1^). This also results in the smallest experimental and calculated Stokes shift (Table 1) across the series, validating the blue shift in the observed emission spectrum. Likewise, to SLA and SALON, SLBO shows high thermal stability, which is consistent with the absence of MLCT absorption bands in the region 25,000–30,000 cm^–1^. One can conclude that as long as valence and MLCT bands are separated by more than 0.3 eV, a thermally stable phosphor should be expected.

## Definition of Geometrical versus Covalency Descriptors

Up to this point of analysis, we have described a computational protocol that provides actual computation of the absorption and emission spectra of a study set of phosphors accompanied by a thorough electronic structure analysis of all the observed spectral features. The protocol has a high predictive ability and is able to explain the energy position and the bandwidth of all the experimentally observed absorption and emission bands. Hence, it serves as a powerful analytical tool in the design and synthesis of new phosphors for analyzing or even predicting the spectroscopic response of known or newly synthesized candidate phosphors. In the same direction, it is also desirable to define descriptors, which could aid the experimental synthesis of candidate phosphors without the need for employing such elaborate calculations in each and every design idea that arrives on the synthesis table. In this sense, a successful definition of descriptors may play a pre-screening role across a massive selection of candidate phosphors toward only those that fulfill the design criteria.

In the Supporting Information, a number of commonly employed descriptors are discussed. It is demonstrated that the energy distribution as well as the intensity mechanism of the absorption and emission process in phosphors goes beyond the geometrical characteristics of the first and second coordination spheres. In contrast, experimental optical band gaps have been employed in order to find linear relationships between absorption and emission energy maxima defining emission color descriptors in a large set of phosphors.^8,29^ However, for a given material, the band gap energies are closely related to the type of employed experimental spectroscopic measurement, the experimental conditions, and the experimental resolution. Hence, typically for a given material, the experimental band gap energy variations range between 0.5 and 1 eV and can reach up to 2–3 eV (e.g., in inorganic semiconductors).^79^ These variations might not always be systematic rendering the definition of experimental band gap energies within a narrow energy window for every candidate-studied system a difficult task. In an alternative scenario, the calculated band gap energies can be used, provided that the employed methodology is carefully calibrated.

**Figure 13:**
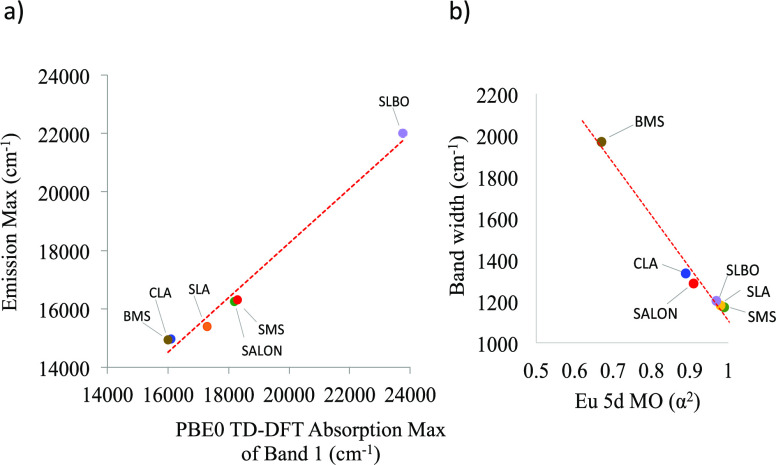
(a) Experimental emission max (cm^–1^) as a function of PBE0 TD-DFT computed absorption max (cm^–1^) of band 1. (b) Experimental emission bandwidth (cm^–1^) as a function of the covalency coefficient *a*^2^ of the acceptor Eu 5d MO in the emission process for SMS (green cycle), BMS (brown cycle), SLA (orange cycle), CLA (blue cycle), SALON (red cycle), and SLBO (purple cycle). The red dotted line displays a linear regression.

It was shown above that the computed PBE0/TD-DFT band gap energies of the SMS and CLA host structures are deviating from experiment and reference DLPNO-STEOM-CCSD calculations by about 0.5–0.8 eV while the computed PBE0/TD-DFT absorption spectra are in very good agreement with respect to the experimental absorption spectral for all the studied phosphors. Hence, in a subsequent step, we investigate the relation of the computed PBE0/TD-DFT absorption energy maximum of band 1 (corresponding to the BG energy) with respect to the experimental emission maximum across the study set of the phosphors (Figure 13a). An excellent linear relation is observed, thus defining a direct emission color descriptor across the study set of the phosphors. The advantage of using computed over experimentally determined optical band gaps is the resolution of the computed band 1, which allows the better definition of the linear relation. At the PBE0/def2-TZVP TD-DFT level, the resulting linear relation reads:
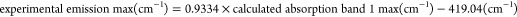
1

In the previous section, it was shown that the bandwidth of the emission band is directly related to the rigidity of the dominating emission process single electron decay. In particular, the nonbonding or the antibonding character of the acceptor Eu 5d MO reached by the absorption process and dominate the respective emission process seems to play a crucial role in the intensity mechanism of the narrow band phosphors. As a measure of this rigidity, we define the coefficient *a*^2^ of the Eu 5d-based MO. Of course, these MO coefficients are not a direct measure of “rigidity”. However, they are a helpful description in this context as they measure the degree of involvement of the Eu center in a covalent bond with its ligands. If there is a degree of covalency in this bond, this also means that there will be a structural distortion upon population or depopulation of the relevant bonding or antibonding orbitals. This gives rise to a structural distortion relative to the ground state, which results in the possibility of a vibronic progression that will in turn broaden the emission band. Thus, as the Eu 4f-based MOs remain essentially nonbonding, the critical quantity is the covalency of the 5d-MO that gets populated in the electronically excited state.

Similar covalency measures have been correlated to a number of spectroscopic properties like metal and ligand hyperfine couplings and zero field splittings in electron paramagnetic resonance (EPR), ligand K-edges in X-ray absorption spectroscopy, and ligand to metal charge transfer (LMCT) intensities.^98−100^ In Figure 13, the linear relation between the experimental emission bandwidth and the Eu 5d coefficient *a*^2^ of the acceptor NTO participating in the emission process is visualized. As seen, the nonbonded acceptor NTO in SMS, SLA, and SLBO obtain *a*^2^ values that are about ∼1. The increased antibonding character of the acceptor NTO in SALON and CLA reduces the *a*^2^ to values that range between 0.8 and 0.9 while they drop to values below 0.6 in the case of BMS in which the acceptor NTO is a ligand-based orbital. As a result, a linear relationship can be identified between the experimental emission bandwidth (in cm^–1^ units) and the computed Eu 5d *a*^2^, which at the PBE0/def2-TZVP TD-DFT level reads:
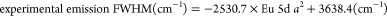
2

Hence, according to the discussion above for a candidate Eu^2+^ phosphor, one only needs to perform a conventional TD-DFT calculation in order to estimate the energy position of the 1st absorption band as well as the Eu 5d *a*^2^ coefficient from an accompanied NTO analysis on this band. This will provide a robust prediction of the expected color and bandwidth of the emission spectrum of the candidate Eu^2+^ phosphor.

## Conclusions

In this work, a computational protocol was developed for the first time that is able to predict the energy position, the shape, and the bandwidth of the absorption and emission spectra of Eu^2+^-doped phosphors. For this purpose, a study set of well-known Eu^2+^-doped nitride, oxo-nitride, and oxide phosphors was chosen, namely, Sr[Mg_3_SiN_4_]:Eu^2+^ (SMS), Ba[Mg_3_SiN_4_]:Eu^2+^ (BMS), Ca[LiAl_3_N_4_]:Eu^2+^ (CLA), Sr[LiAl_3_N_4_]:Eu^2+^ (SLA), Sr[Al_2_Li_2_O_2_N_2_]:Eu^2+^ (SALON), and SrLi_2_[Be_4_O_6_]:Eu^2+^ (SLBO). This set of phosphors contains a broad variety of energy shifts of the absorption and emission spectral features as well as the emission bandwidths. The construction of cluster models was performed in the framework of the embedded cluster approach. The size of the designed cluster models converged rapidly with respect to the band gap of the host ligand families computed at the STEOM-DLPNO-CCSD and TD-DFT levels of theories as well as the absorption spectra shapes computed at the TD-DFT level of theory.

Prior to the spectra computations, a detailed geometrical and electronic structure analysis was performed, which helped to identify those factors that influence the intensities and the energy distribution of the absorption and emission spectra in terms of (1) crystal field strengths, (2) the coordination environment around the Eu centers, (3) the Stokes shift variations, and (4) the nature of the single electron excitations or electron decays dominating the absorption and emission processes.

In a next step, the shapes of the absorption and the fluorescence spectra of the family of the chosen Eu^2+^-doped phosphors were computed at the TD-DFT level in the framework of the excited state dynamics (ESD) approach. The excellent agreement between theory and experiment allowed a quantitative electronic structure analysis in the framework of natural transition orbitals analysis. It was shown that the energy position and the bandwidth of the emission band are influenced by the rigidity of the electron decay processes dominating the emission spectral feature. In particular, the nonbonding character of the acceptor Eu 5d NTO is responsible for the narrow band emission and the small Stokes shifts of the emission bands in SMS and SLBO. On the contrary, as the antibonding character of the acceptor Eu 5d NTO increases (the cases of SLA, CLA, and SALON), the bandwidth as well as the Stokes shift of the emission band increases. In BMS, the ligand-based antibonding σ*(Si-2s, N-2p) MO is stabilized below the Eu-5d MOs. As a result, the rigidity of the electron decay dominating the emission process is altered, leading to an increase in both the emission bandwidth and the Stokes shift. The effect is strong and is able to overcome the expectations from the crystal field strength. As a result, a red shift broad emission spectrum is observed. It should be mentioned that such broadening effects owning to the low-lying MLCT transitions are also referred to as “trapped exciton emission” or “anomalous emission” and have been observed in other phosphor materials.^101^ As a measure of the thermal stability of the studied Eu^2+^-doped phosphors, the energy separation between the valence and MLCT absorption bands was defined. In principle, an energy separation that is above 0.3 eV points to thermally stable Eu^2+^-doped phosphors as in the case of SLA, SALON, and SLBO. The above-presented protocol was found to perform equally well in all the studied phosphors. Hence, in a final step of the analysis, it was employed to identify a uniform set of descriptors that are able to estimate both the energy position and the bandwidth of the emission bands of newly designed Eu^2+^-doped candidate materials. It was found that the energy position of the 1st computed band relates linearly with the energy position of the experimental emission band. Meanwhile, analysis of the nature of the one electron excitation dominating this band revealed that the covalency coefficient Eu 5d *a*^2^ relates also linearly with the bandwidth of the emission band. We foresee that it will be possible in the future to employ these descriptors for pre-screening large datasets of Eu^2+^-doped phosphor candidates for application in LEDs.

Ongoing research in our laboratories is focusing on further understanding the intensity mechanism as well as the quantum efficiency of narrow band phosphors in terms of multiplet effects and vibronic couplings through extension of the developed computational protocol. In parallel, a real-life application of the identified descriptors is performed in an effort to possibly identify new materials that can serve as narrow band phosphors with tailored properties.

## Supporting Information Available

Further details regarding the probable Eu^2+^-doped centers, rigidity factors, and absorption and emission spectra and relaxation times/rates of the studied phosphors (PDF)
