# Supplementary material for: Electronic and Optical Properties of Eu2+-Activated Narrow-Band Phosphors for Phosphor-Converted Light-Emitting Diode Applications: Insights from a Theoretical Spectroscopy Perspective
Source: J Am Chem Soc. 2022 Apr 26;144(18):8038–53. doi: 10.1021/jacs.2c00218 (PMC9100680; doi:10.1021/jacs.2c00218)
Supplement: Supplementary file 1 — ja2c00218_si_001.pdf [file ja2c00218_si_001.pdf]

Electronic and Optical Properties of Eu^{2+}-activated Narrow-Band Phosphors for pc-LED Applications. Insights from a Theoretical Spectroscopy Perspective

Rami Shafei, Dimitrios Maganas,* Philipp Jean Strobel, Peter J. Schmidt,

Wolfgang Schnick* and Frank Neese*

Supporting Information

Table of Contents

I. Tables **3**

Table S1. **3**

Table S2. **4**

Table S3. **5**

Table S4. **5**

II. Figures **6**

Figure S1. **6**

Figure S2. **7**

III. Descriptors in phosphors **8**

IV. References **9**

I. Tables

Table S1.

Detailed description of the employed clusters within the embedded cluster approach in terms of 1) the QC and HF layers compositions, 2) the number of atoms in the ECP region, 3) the number of atoms in the PC region and 4) the converged CHELPG charges.

Phosphor	QC+HF	n(cECP)	n(PC)	CHELPG Charges
$Sr[Mg_3SiN_4]:Eu^{2+}$	$\{[EuMg_9Si_3N_{24}]^{40-} + [Sr_2Mg_{12}Si_3]^{40+}\}^0$	186	42130	q(Sr, Mg , Si, N)
SMS	$\{[EuSrMg_{15}Si_5N_{36}]^{54-} + [Sr_2Mg_{17}Si_4]^{54+}\}^0$	222	42093	(2.07, 2.03, 3.56, -2.81)
	$\{[EuSr_2Mg_{21}Si_7N_{48}]^{68-} + [Sr_2Mg_{22}Si_5]^{68+}\}^0$	267	41878	
$Ba[Mg_3SiN_4]:Eu^{2+}$	$\{[EuMg_9Si_3N_{24}]^{40-} + [Ba_2Mg_{13}Si_3]^{40+}\}^0$	186	34464	q(Sr, Mg, N)
BMS	$\{[EuBaMg_{15}Si_5N_{36}]^{54-} + [Ba_2Mg_{17}Si_4]^{54+}\}^0$	222	34407	(2.20, 2.26, -2.74)
	$\{[EuBa_2Mg_{21}Si_7N_{48}]^{68-} + [Ba_2Mg_{22}Si_5]^{68+}\}^0$	460	34148	
$Ca[LiAl_3N_4]:Eu^{2+}$	$\{[EuLi_3Al_9N_{24}]^{40-} + [Ca_2Li_3Al_{11}]^{40+}\}^0$	186	46838	q(Ca, Li , Al, N)
CLA	$\{[EuCaLi_5Al_{15}N_{36}]^{54-} + [Ca_2Li_8Al_{14}]^{54+}\}^0$	222	46781	(1.76, 1.36, 2.70, -2.81)
	$\{[EuCa_2Li_7Al_{21}N_{48}]^{68-} + [Ca_2Li_7Al_{19}]^{68+}\}^0$	258	46724	
$Sr[LiAl_3N_4]:Eu^{2+}$	$\{[EuSrLi_5Al_{15}N_{36}]^{54-} + [Sr_2Li_8Al_{14}]^{54+}\}^0$	218	42600	q(Sr, Li , Al, N)
SLA	$\{[EuSr3Li_{10}Al_{26}N_{60}]^{84-} + [SrLi_{10}Al_{24}]^{84+}\}^0$	494	42482	(2.00, 0.94, 2.93, -2.87)
$Sr[Al_2Li_2O_2N_2]:Eu^{2+}$	$[EuLi_6Al_6O_{12}N_{12}]^{34-} + [SrLi_8Al_8]^{34+}\}^0$	186	39486	q(Sr, Li , Al, O, N)
SALON	$\{[EuSrLi_{10}Al_{10}O_{18}N_{18}]^{46-} + [SrLi_{11}Al_{11}]^{46+}\}^0$	222	39429	(1.60, 1.16, 2.74, -1.78, -2.90)
	$\{[EuSr_2Li_{14}Al_{14}O_{24}N_{24}]^{58-} + [SrLi_{14}Al_{14}]^{58+}\}^0$	438	39372	
$SrLi_2[Be_4O_6]:Eu^{2+}$	$\{[EuLi_4Be_{12}O_{32}]^{34-} + [Li_4Be_{15}]^{34+}\}^0$	327	43016	q(Sr, Li , Be, O)
SLBO	$\{[EuSrLi_8Be_{20}O_{52}]^{52-} + [SrLi_6Be_{21}]^{52+}\}^0$	443	42867	(2.10, 1.00, 1.31, -1.94)
	$\{[EuSr_2Li_{12}Be_{28}O_{72}]^{70-} + [SrLi_{10}Be_{30}]^{70+}\}^0$	559	42718	

Table S2.

Experimental host volume (Å^3) and average Avg. Eu^{2+}-N bond distance (Å) of cation substituted EuL_8 cuboids and empty channels EuL_6 octahedra (L = N or O) in BMS, SMS, CLA, SLA, SALON and SLBO phosphors.

Phosphor	L	Volume		Avg. Eu^{2+}-N	
		EuL_8	EuL_6	EuL_8	EuL_6
$Ba[Mg_3SiN_4]:Eu^{2+}$ BMS	N	39.54	20.01	2.90	2.48
$Sr[Mg_3SiN_4]:Eu^{2+}$ SMS	N	36.95	19.72	2.86	2.46
$Ca[LiAl_3N_4]:Eu^{2+}$ CLA	N	31.88	15.82	2.75	2.33
$Sr[LiAl_3N_4]:Eu^{2+}$ SLA	N	34.00	17.58	2.80	2.41
$Sr[Al_2Li_2O_2N_2]:Eu^{2+}$	N	30.41	--	2.76	--
SALON	O		20.48	2.66	2.53
$SrLi_2[Be_4O_6]:Eu^{2+}$	O	28.81	10.84	2.60	2.06
SLBO				2.80	

Table S3.

Computed $FWHM_{300K}$, reorganization energy and Huang–Rhys factor S for BMS, SMS, CLA, SLA, SALON and SLBO phosphors.

Phosphor	$FWHM_{300K}$ cm^{-1}[nm]	Reorganization Energy (cm^{-1})	S
$Ba[Mg_3SiN_4]:Eu^{2+}$ BMS	2015 [90]	300.44	0.360
$Sr[Mg_3SiN_4]:Eu^{2+}$ SMS	1150 [43]	70.29	0.084
$Ca[LiAl_3N_4]:Eu^{2+}$ CLA	1340 [60]	28.40	0.035
$Sr[LiAl_3N_4]:Eu^{2+}$ SLA	1140 [50]	7.85	0.010
$Sr[Al_2Li_2O_2N_2]:Eu^{2+}$ SALON	1220 [46]	23.72	0.029
$SrLi_2[Be_4O_6]:Eu^{2+}$ SLBO	1220 [25]	8.58	0.012

Table S4.

Calculated luminescence rates, r_{flu} (s^{-1}) and relaxation times $\tau_{flu.}$ (s) for the study set of the Eu^{2+} doped phosphors within Franck Condon (FC) and with and without Herzberg Teller (HT) corrections.

Phosphor	Doping site	FC		FC/HT	
		$r_{flu.}$ (s^{-1})	$\tau_{flu.}$ (s)	$r_{flu.}$ (s^{-1})	$\tau_{flu.}$ (s)
$Ba[Mg_3SiN_4]:Eu^{2+}$ BMS	Ba^{2+}	$1.5x10^4$	$6.7\ x10^{-5}$	$1.5x10^5$	$1.9\ x10^{-6}$
$Sr[Mg_3SiN_4]:Eu^{2+}$ SMS	Sr^{2+}	$1.4x10^5$	$7.1\ x10^{-6}$	$1.4x10^9$	$1.1\ x10^{-10}$
$Ca[LiAl_3N_4]:Eu^{2+}$ CLA	Ca^{2+}	$6.9x10^4$	$1.5\ x10^{-5}$	$6.9x10^{10}$	$3.3\ x10^{-11}$

Sr[LiAl$_3$N$_4$]:Eu^{2+} SLA	Sr^{2+}	1.3x10^5	7.7 x10^{-6}	1.3x10^9	1.4 x10^{-10}
Sr[Al$_2$Li$_2$O$_2$N$_2$]:Eu^{2+} SALON	Sr^{2+}	1.4x10^3	7.1 x10^{-4}	1.4x10^8	2.2 x10^{-9}
SrLi$_2$[Be$_4$O$_6$]:Eu^{2+} SLBO	Sr^{2+}	1.3x10^4	7.7 x10^{-5}	1.3x10^8	1.6 x10^{-9}

II. Figures

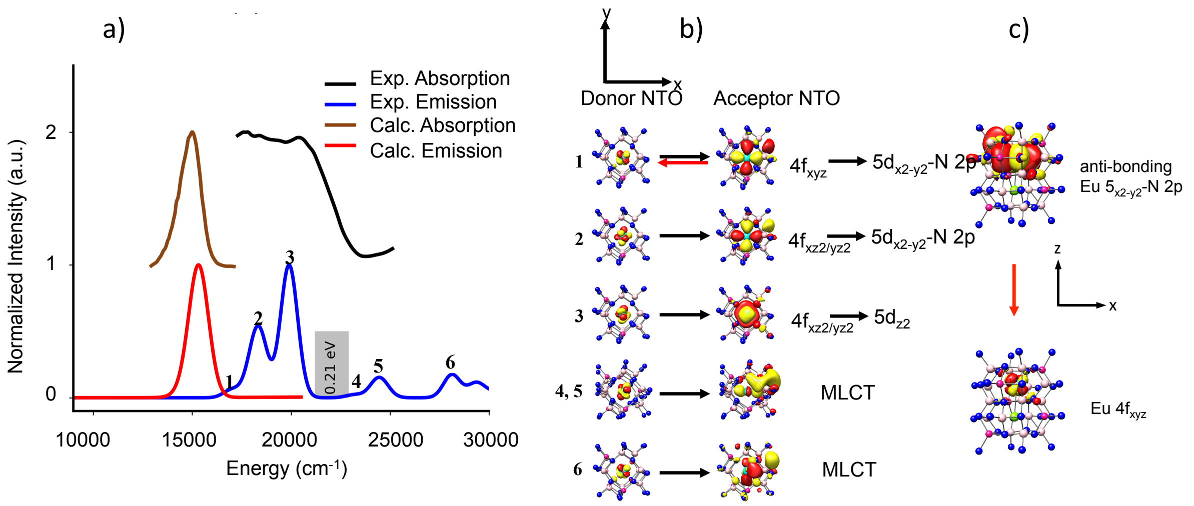

Figure S1.

a) CLA experimental (black), calculated TDDFT/PBE0 absorption (blue, light blue) spectra and experimental (brown), TDDFT/PBE0/ESD calculated (red) emission spectra. b) NTO analysis of the relevant bands in absorption spectra and c) the 1st transition responsible for emission upon relaxation.

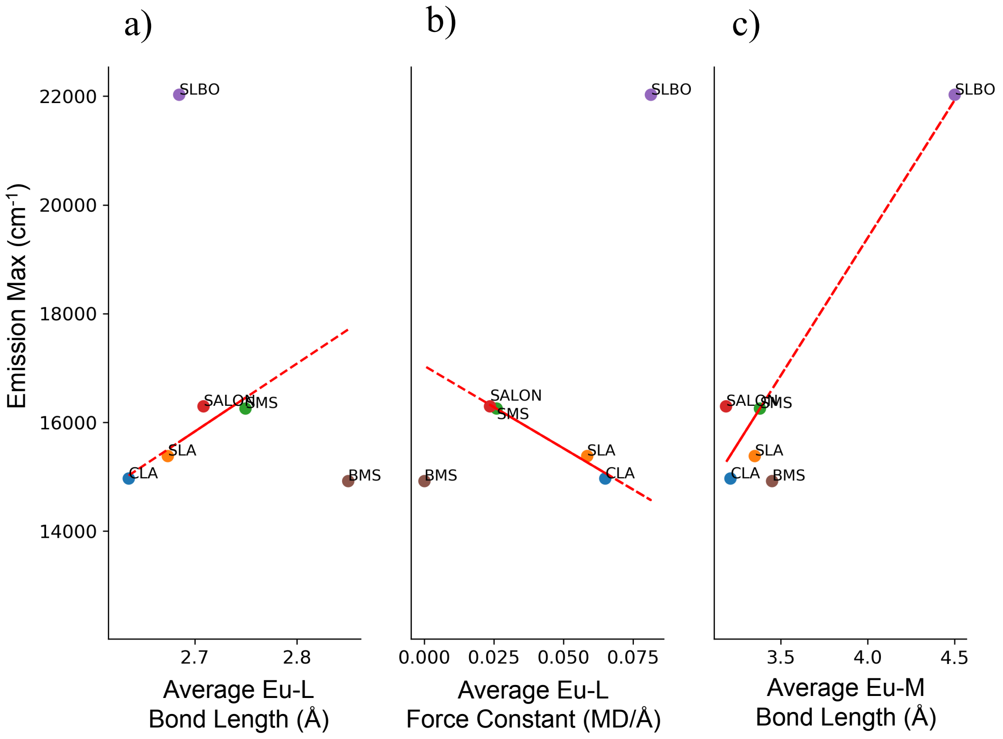

Figure S2.

Experimental emission Max (cm^{-1}) as a function of a) the average bond length Eu – L (Å), b) the average force constant Eu – L (MD/Å) and c) the average bond length Eu – M (Å) in SMS (green cycle), BMS (brown cycle), SLA (orange cycle), CLA (blue cycle), SALON (red cycle) and SLBO (purple cycle). Red dot is a linear regression.

III. Descriptors in phosphors

Experimental optical band gaps as well as the energy splitting of the f-orbitals define two widely used descriptors. In fact, as it was shown in Scheme 1, optical band gaps can be used to predict the energy position of the emission band and define the color of the phosphor. Recently, experimental band gaps have been used as descriptors in the framework of machine learning techniques[1-2] Similarly, a quantitative descriptor refers to the energy splitting between the two highest Eu f-based MOs. Factually, it has been shown that an energy separation by more than 0.1 eV is necessary to achieve emission with narrow bandwidth.[3] This is based on the idea that large energy splitting in these MOs will prevent multiple overlapping relaxation transitions giving homogenous emission with narrow band. Unfortunately, none of the above descriptors is able to properly probe the chemical environment around the doped Eu^{2+} across the phosphors. Hence such descriptors cannot capture the case of anomalous emission of BMS or the Stokes shift variation across the different phosphors of the study set. It was shown above that the energy position and bandwidth of the emission spectrum in phosphors is directly related to the metal-ligand covalency around the Eu^{2+} centers. In the following we will develop descriptors that are based on the metal-ligand chemical environment of these systems.

First, the relation of the coordination environment around the Eu^{2+} centers with the experimental emission maximum across the set of the chosen phosphors is investigated. The results are shown in Figure S2. As is shown, a linear relation between the average bond length Eu-L, L=N, O is obtained for the series SMS, CLA, SLA and SALON . As described in Figure 1 this behavior reflects the fact that a decrease of the crystal field strength is associated with a blue shift in the emission maximum. However, such a relation is still not sensitive enough to predict the red shift of BMS as well as the blue shift of SLBO. Computation of the respective Eu-L, L=N, O average force constants across the series shows the exact same behavior (Figure S2b) which might be expected based on Badger's rule.[4] By contrast, some degree of linear relation involving the entire study set of the phosphors is observed between the average bond length Eu-M, M=Sr, Ca, Ba of the Eu center and the host ligand counter ion (Figure S2c). However, none of these descriptors is entirely satisfactory.

IV. References

(1) Zhuo, Y.; Mansouri Tehrani, A.; Oliynyk, A. O.; Duke, A. C.; Brgoch, J., Identifying an efficient, thermally robust inorganic phosphor host via machine learning. *Nat. Commun.* **2018,** *9*, 4377.

(2) Zhuo, Y.; Mansouri Tehrani, A.; Brgoch, J., Predicting the Band Gaps of Inorganic Solids by Machine Learning. *J. Phys. Chem. Let.* **2018,** *9*, 1668-1673.

(3) Wang, Z. B.; Chu, I. H.; Zhou, F.; Ong, S. P., Electronic Structure Descriptor for the Discovery of Narrow-Band Red-Emitting Phosphors. *Chem. Mater.* **2016,** *28*, 4024-4031.

(4) Badger, R. M., The Relation Between the Internuclear Distances and Force Constants of Molecules and Its Application to Polyatomic Molecules. *J. Chem. Phys.* **1935,** *3*, 710-714.
